# Identifying novel proteins for migraine by integrating proteomes from blood and CSF with genome‐wide association data

**DOI:** 10.1111/cns.14817

**Published:** 2024-06-19

**Authors:** Peng‐Peng Niu, Rui Zhang, Chan Zhang, Shuo Li, Yu‐Sheng Li

**Affiliations:** ^1^ Department of Neurology The First Affiliated Hospital of Zhengzhou University Zhengzhou China

**Keywords:** causal relationship, co‐localization, Mendelian randomization, migraine, plasma protein, therapeutic targets

## Abstract

**Background:**

Proteome‐wide Mendelian randomization studies have been increasingly utilized to identify potential drug targets for diseases. We aimed to identify potential therapeutic targets for migraine and its subtypes through the application of Mendelian randomization and co‐localization analysis methods.

**Methods:**

We utilized *cis‐*protein quantitative trait loci data for 1378 plasma proteins available from two studies with 7213 individuals and 35,559 individuals, respectively. Summary data for migraine and its subtypes were obtained from a genetic study involving up to 1,339,303 individuals. Proteins that passed both the discovery and validation Mendelian randomization analysis, sensitivity analysis, heterogeneity test, and pleiotropy test, were associated with ≥2 outcomes, and received strong support from co‐localization analysis (PP.H4.abf ≥0.80) and were classified as tier 1 proteins.

**Results:**

We identified three tier 1 proteins (LRP11, ITIH1, and ADGRF5), whose genes have not been previously identified as causal genes for migraine in genetic studies. LRP11 was significantly associated with the risk of any migraine (OR [odds ratio] = 0.968, 95% CI [confidence interval] = 0.955–0.981, *p* = 1.27 × 10^−6^) and significantly/suggestively associated with three migraine subtypes. ITIH1 was significantly associated with the risk of any migraine (OR = 1.044, 95% CI = 1.024–1.065, *p* = 1.08 × 10^−5^) and migraine with visual disturbances. ADGRF5 was significantly associated with the risk of any migraine (OR = 0.964, 95% CI = 0.946–0.982, *p* = 8.74 × 10^−5^) and suggestively associated with migraine with aura. The effects of LRP11 and ADGRF5 were further replicated using cerebrospinal fluid protein data. Apart from ADGRF5, there was no evidence of potential adverse consequences when modulating the plasma levels. We also identified another four proteins (PLCG1, ARHGAP25, CHGA, and MANBA) with no potential adverse consequences when modulating the plasma levels, and their genes were not reported by previous genetic studies.

**Conclusions:**

We found compelling evidence for two proteins and suggestive evidence for four proteins that could be promising targets for migraine treatment without significant adverse consequences. The corresponding genes were not reported in previous genetic studies. Future studies are needed to confirm the causal role of these proteins and explore the underlying mechanisms.

## Graphical abstract

With the application of Mendelian randomization and co‐localization analysis methods, we found compelling indications for two proteins (LRP11 and ITIH1) and suggested indications for four proteins (PLCG1, ARHGAP25, CHGA, and MANBA), which might be promising targets for migraine treatment without obvious adverse consequences.

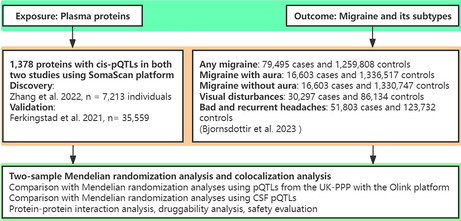

## 1 BACKGROUND

Migraine is a prevalent and debilitating neurological disorder that affects a significant portion of the global population.^1^, ^2^ It is characterized by recurring episodes of severe headache accompanied by various symptoms, such as nausea, sensitivity to light and sound, and in some cases, aura.^3^ The exact underlying mechanisms and causes of migraine are not fully understood, which poses challenges in developing effective treatment strategies for this condition. Despite the groundbreaking advancements facilitated by calcitonin gene‐related peptide monoclonal antibodies, a notable proportion of patients continue to endure an inadequate treatment response.^4^, ^5^

Previous studies have highlighted the association between alterations in blood plasma proteins and the occurrence of migraines.^6^, ^7^, ^8^, ^9^, ^10^ Several studies have identified specific plasma proteins that are dysregulated in individuals with migraines compared to healthy controls. For example, elevated levels of certain inflammatory markers, such as C‐reactive protein, interleukin‐6, and tumor necrosis factor‐alpha, and adipocytokines of leptin and adiponectin have been observed in migraine patients.^11^, ^12^, ^13^ In addition, non‐inflammatory proteins,^7^, ^10^ such as the regulatory proteins involved in maintaining vascular integrity, have also been observed to be linked with migraines.^7^

Considering the emerging understanding of the role of plasma proteins and inflammatory factors in migraine, there is a compelling need to further investigate these associations using advanced analytical techniques. In recent years, with the availability of large genetic studies on various traits including plasma proteins and diseases, Mendelian randomization (MR) analysis has emerged as a powerful means for identifying causal links between traits and diseases, as well as between potential drug targets and diseases.^14^, ^15^, ^16^, ^17^, ^18^ By leveraging genetic variants as instrumental variables, MR analysis can provide valuable insights into the causal effects of specific proteins on disease outcomes.^19^

In this study, we employed proteome‐wide MR study to comprehensively explore the causal relationship between plasma proteins and migraine and its subtypes. Moreover, we conducted co‐localization analyses to reinforce the findings obtained from MR analyses.

## 2 METHODS

**FIGURE 1:**
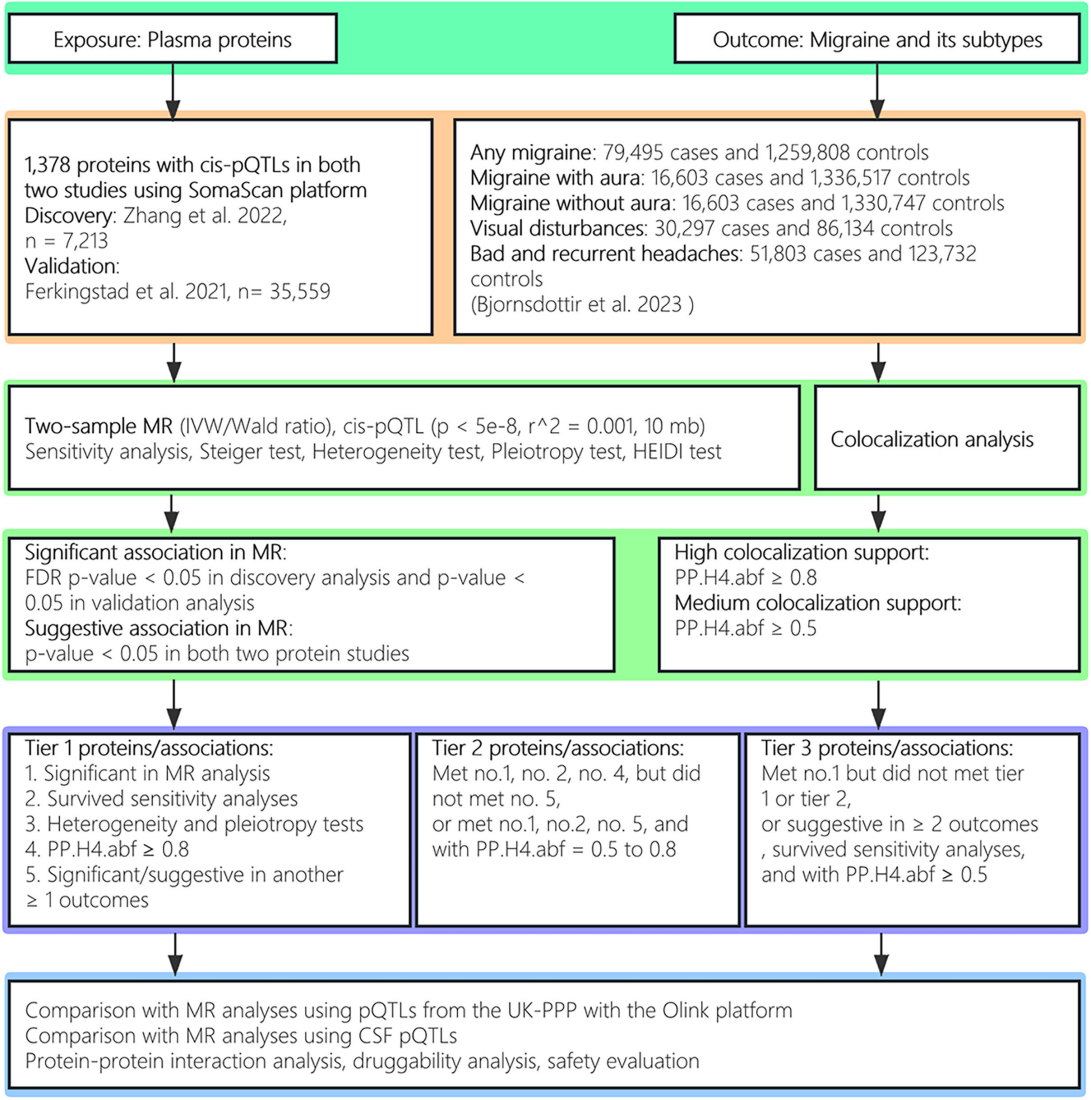
Design of the present study. The three core assumptions for the Mendelian randomization analysis were as follows. (1) Relevance: The genetic variants used as instruments are associated with the exposure variable. (2) Independence: The genetic variants are independent of potential confounders that may affect the outcome variable. (3) Exclusion restriction: The genetic variants influence the outcome variable only through their effect on the exposure variable and not through any alternative pathways. CSF, cerebrospinal fluid; FDR, false discovery rate; HEIDI, heterogeneity in dependent instruments; IVW, inverse variance‐weighted; MR, Mendelian randomization; pQTL, protein quantitative trait loci.

Figure 1 shows the design of the present study. Table S1 shows the datasets included in the present study.

### 2.1 Instrument selection

#### 2.1.1 Plasma protein quantitative trait loci (pQTL)

We utilized plasma pQTL data from two separate studies conducted by Zhang et al. and Ferkingstad et al. to generate genetic instruments.^20^, ^21^ Summary‐level data, generated by Zhang et al.,^20^ on 4483 plasma proteins from 7213 individuals of European American descent, who participated in the Atherosclerosis Risk in Communities study, were utilized for the discovery analysis. This dataset was chosen as it does not have any sample overlap with our outcome dataset. Summary‐level data, generated by Ferkingstad et al.,^21^ on 4719 plasma proteins from a cohort of 35,559 individuals from Iceland, were employed for the replication analysis. Both studies utilized the SomaScan multiplex aptamer assay (version 4) to measure plasma protein levels. The significant overlap of proteins between these two studies provides us with the opportunity to verify and validate as many results as possible, ensuring a robust analysis.

Our research primarily centers on *cis‐*pQTLs for conducting the MR analysis. These loci have demonstrated higher reproducibility across diverse proteomic platforms compared to *trans‐*pQTLs. Moreover, *cis‐*pQTLs are less susceptible to the influence of horizontal pleiotropy, which can present additional challenges in MR analyses.^14^, ^20^

The criterion for defining *cis‐*pQTLs, as used by Zhang et al., involves a mapping window spanning 500 kb around target protein‐coding genes. We have also adopted this criterion in our study, which has been widely embraced by other studies as well.^15^

To acquire independent genetic instruments, a multi‐step process was followed. Initially, all single‐nucleotide polymorphisms (SNPs) within the *cis‐*region were selected based on a genome‐wide *p*‐value threshold of <5 × 10^−8^. Subsequently, palindromic SNPs (i.e., A/T and G/C) displaying intermediate allele frequency (0.4–0.6) were excluded. Additionally, SNPs located in proximity to the human leukocyte antigen region (chr6:29–34 Mb) were also removed from consideration due to the region's complex linkage disequilibrium structure, which makes it susceptible to horizontal pleiotropy and violations of the MR assumptions.^22^, ^23^ Finally, to obtain independent genetic instruments, we conducted a clumping procedure using an *r* ^2^ threshold of 0.001 and a physical window of 10 MB. For the clumping procedure, we utilized the European reference panel from the 1000 Genome Project Phase 3, with a minimum minor allele frequency of 0.01, as our reference panel. The instruments used in this study had a minimum *F* statistic of 29.7, surpassing the recommended threshold of 10 (Table S2).

#### 2.1.2 Plasma protein pQTLs using another platform

In a recent genetic study, a comparison was made between two affinity‐based platforms: Olink Explore 3072, which used data from the UK Biobank, and SomaScan v4, which was generated in Iceland.^24^ The findings revealed a moderate correlation between the two platforms. In addition, it was observed that a significant number of proteins exhibited genomic associations that differed between the platforms. In light of this, we conducted MR analysis using plasma pQTL data from the UK Biobank Pharma Proteomics Project to assess the consistency of associations observed with the SomaScan platform (Table S3).^25^

### 2.2 Cerebrospinal fluid (CSF) pQTL

We conducted MR analysis using CSF pQTL data to evaluate the consistency of associations observed with plasma pQTL data (Table S4). Cruchaga et al.^26^ have performed a largest‐to‐date CSF pQTL atlas by analyzing 7028 proteins in 3107 samples. CSF proteins were measured using the SomaScan platform, which is same as the plasma pQTLs we used. It is important to note that a significant portion of the dataset used in this study consisted of individuals with Alzheimer's disease. However, the analysis revealed that the identified pQTLs remained consistent across different disease states.

### 2.3 Migraine datasets

Bjornsdottir et al.^27^ recently published a meta‐analysis by combining large genome wide association study (GWAS) datasets from European populations, which included approximately 1.2 million individuals. The datasets were sourced from Iceland (deCODE Genetics), Denmark (Copenhagen Hospital Biobank (CHB) and Danish Blood Donor Study (DBDS)), the United Kingdom (UK Biobank), the United States (Intermountain Health), Norway (the Hordaland Health Study (HUSK)), and Finland (FinnGen).

The study included a total of 79,495 individuals with clinically defined overall migraine, 16,603 individuals with clinically defined migraine with aura (MA), and 11,718 individuals with clinically defined migraine without aura (MO). The control population consisted of 1,259,808 individuals for any migraine, 1,336,517 individuals for MA, and 1,330,747 individuals for MO. In addition, there were 51,803 individuals with self‐reported bad and recurrent migraine headaches (BRH) and 123,732 individuals without BRH. Furthermore, there were 30,297 individuals with self‐reported visual disturbances (VD) before or near headaches, and 86,134 individuals without VD.

### 2.4 Proteome‐wide two‐sample MR analysis

We employed various MR methods to assess causal relationships using the “TwoSampleMR” R package. For SNPs that were not found in the outcome datasets, a proxy single‐nucleotide polymorphism (SNP) with an *r* ^2^ value of ≥0.8 was used, if it was available. The outcome datasets were transformed to “GWAS vcf” format using the “create_vcf” function in the “gwasvcf” R package. Proxy SNP was found and extracted using the “query_gwas” function in the “gwasvcf” R package.

The discovery and validation MR analyses used SNPs with linkage disequilibrium *r* ^2^ < 0.001. The MR method used was the Wald ratio method for exposures with a single instrumental SNP, and the random‐effects inverse variance‐weighted (IVW) method for exposures with multiple instrumental SNPs. The estimation of the Wald ratio method was obtained by dividing the instrument‐outcome estimation by the instrument‐exposure estimation. The estimation of the IVW method was obtained by combining the Wald ratio estimates in a random‐effects meta‐analysis. In this analysis, the weight assigned to each ratio was the inverse of the variance of the SNP‐outcome estimation.

Furthermore, to ensure robustness and sensitivity of our findings, we conducted additional analyses using different MR methods including MR–Egger method, weighted median method, and weighted mode method, if there were a sufficient number of SNPs available.

To further validate the observed associations, we conducted generalized summary‐data‐based MR (GSMR) analyses using the GSMR R package.^28^ The GSMR method provides greater statistical power in comparison with the previously mentioned standard IVW methods. This is achieved by considering the sampling variance in both instrument‐exposure and instrument‐outcome associations. Moreover, the GSMR method takes into account the correlation of linkage disequilibrium between variants and incorporates an outlier analysis to identify and eliminate any outliers. The final GSMR analysis only included SNPs that passed the outlier analysis. We employed a relaxed clump criterion (*r* ^2^ < 0.1) to ensure an adequate number of SNPs for our analysis. Since the GSMR method was considered as a validation method, we included SNPs that were reported in at least two of the five outcome cohorts. Additionally, we only included SNPs with low heterogeneity, which was defined as having a heterogeneity *p*‐value greater than 0.05 and a heterogeneity *I*‐squared statistic lower than 50%. This selection criterion helped to ensure that the chosen SNPs were consistent across multiple cohorts and had minimal heterogeneity. Heterogeneity test and pleiotropy test were performed by using SNPs included in GSMR method, when there were a sufficient number of SNPs available. For pQTLs with insufficient SNPs to perform the GSMR analysis, we performed IVW analysis using SNPs with linkage disequilibrium *r* ^2^ < 0.1. The correlation of linkage disequilibrium between SNPs were considered by using “MendelianRandomization” R package.

In order to examine the directionality of the MR estimations, we performed an MR Steiger test.^29^ We also conducted Steiger tests for each individual SNP, and the results indicated that all instrumental SNPs exhibited a greater effect in pQTLs than in the outcome (Steiger test *p* values ≤0.020).

We also performed reverse MR to examine the causal effects of migraine on identified pQTLs. For migraine subtypes, due to limited number of SNPs with *p* value <5 × 10^−8^, we relaxed the threshold to <1 × 10^−5^ to select instruments.

For results of MR analyses, we defined significant association as false discovery rate (FDR) corrected *p*‐value in discovery analysis <0.05 and *p*‐value <0.05 in validation analysis. We defined suggestive association as *p*‐value <0.05 in both two pQTL studies.

### 2.5 SNPs with potential pleiotropic effects

We conducted a query of the PhenoScanner V2 database using the “phenoscanner” R package to identify potentially pleiotropic SNPs associated with confounding variables at a genome‐wide *p*‐value threshold of <5 × 10^−8^.^30^, ^31^ The confounding variables were identified through a comprehensive literature review, which including factors such as coffee intake, smoking,^32^ blood pressure traits, psychological traits, blood lipid traits,^33^ diabetes, hematological parameters,^34^ and kidney function traits.^35^ We performed a sensitivity analysis by excluding SNPs with pleiotropic effects. Initially, we examined the effects of all SNPs (without clumping) on the outcomes. Only SNPs showing low heterogeneity, as defined previously, and SNPs with a *p*‐value >5 × 10^−8^ for the outcome were included in the analysis. Subsequently, any pleiotropic SNPs that associated with above mentioned cofounders were carefully excluded from further analysis. In order to identify instrumental SNPs for subsequent MR sensitivity analysis on each specific protein, we applied a clumping procedure with an *r* ^2^ threshold of 0.001 and a physical window of 10 MB.

### 2.6 Heterogeneity in dependent instruments test

For associations supported by MR analysis, we performed heterogeneity in dependent instruments (HEIDI) test to detect whether the association patterns across the *cis‐*region are homogeneous or not. A heterogeneous pattern indicates that there might be two or more genetic variants in linkage disequilibrium affecting the exposure and outcome independently.^36^ We used a command line based tool of “smr (version 1.3.1)” (https://yanglab.westlake.edu.cn/software/smr/) to perform the HEIDI test. As recommend by the authors, at least 3 SNPs and up to 20 SNPs were included in the test. The SNPs included in the test should have a maximal *p* value of 1.57 × 10^−3^. A *p* value of <0.05 of the HEIDI test suggests support for the presence of a shared single causal variant between pQTL and migraine.

### 2.7 Bayesian co‐localization analysis

For associations supported by MR analysis, we employed Bayesian co‐localization analysis to determine whether two traits are influenced by the same or distinct causal variants within a specific genomic region.^37^ The “coloc” R package (http://cran.r‐project.org/web/packages/coloc) was utilized for performing the co‐localization analysis. Default parameters were used for prior probabilities, including a prior of 1 × 10^−4^ for p1 and p2, and a prior probability of 1 × 10^−6^ for p12. The analysis provides five hypotheses (H0, H1, H2, H3, and H4), each accompanied by its corresponding posterior probability. Our main focus was on the posterior probability of the fifth hypothesis (H4). A posterior probability of H4 (PP.H4.abf) ≥ 80% suggests strong support for the presence of a shared single causal variant between pQTL and migraine. A posterior probability of H4 (PP.H4.abf) ≥ 50% indicates moderate support.

It is important to note that the standard co‐localization analysis performed by “coloc” assumes the existence of only one single causal variant for both traits in any given genomic region. Additionally, we conducted co‐localization analysis using the Sum of Single Effects approach, which allows simultaneous evaluation of evidence for association at multiple causal variants.^38^

Co‐localization results typically reach significance only when there is strong support for associations of SNPs with both traits. Conversely, a nonzero estimate obtained from MR analysis may still be observed, even if the associations of SNPs with the outcome are only nominally significant.^39^

### 2.8 Protein–protein interaction, druggability evaluation, and safety evaluation

To explore the potential interactions between the identified proteins, we constructed a comprehensive protein–protein interaction network using the STRING database (https://string‐db.org/). This database includes both experimentally validated and predicted protein interactions, providing valuable insights into the functional relationships among proteins. By analyzing the protein–protein interaction network, we aimed to uncover the underlying molecular mechanisms and pathways associated with our target proteins.

We conducted an extensive evaluation of the identified proteins as potential therapeutic targets. To accomplish this, we employed multiple databases, including DGIdb (https://www.dgidb.org/) and ChEMBL (https://www.opentargets.org/). These databases integrate information from various sources, such as drug‐gene interactions, gene function annotations, text mining, and expert curation. By leveraging these resources, we prioritized the druggability of our target proteins based on their potential interactions with known drugs.

We next performed target safety assessments to assess the potential unintended adverse consequences when modulating the target. We conducted MR analyses using the identified proteins as exposures and several common cancers and cardiovascular diseases as outcomes.

## 3 RESULTS

### 3.1 Proteome‐wide two‐sample MR analysis

**FIGURE 2:**
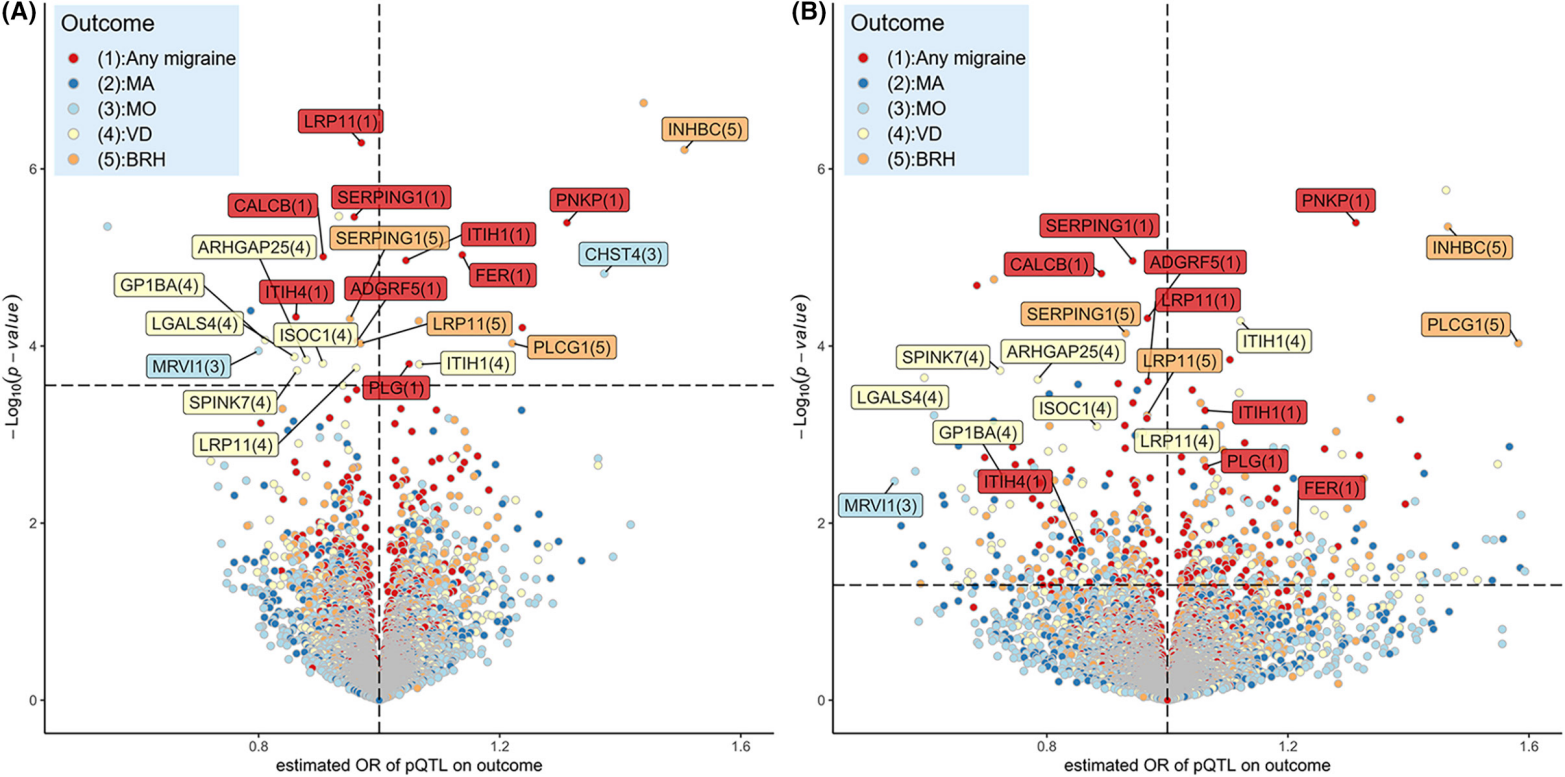
Volcano plots of the MR results for plasma proteins on the risk of migraine and its subtypes. (A) The MR results using discovery pQTL data (ARIC; Zhang et al., 2022). (B) The MR results using validation pQTL data (decode; Ferkingstad et al., 2021). The dashed horizontal black line in A corresponds to a *p*‐value of 2.14E‐4 (FDR‐corrected *p*‐value of 0.05). The dashed horizontal black line in B corresponds to a *p*‐value of 0.05. Proteins that showed a significant association using discovery pQTL data and a suggestive association using validation pQTL data, and exhibited the same effect direction were labeled. BRH, bad and recurrent migraine headaches; FDR, false discovery rate; MA, migraine with aura; MO, migraine without aura; MR, Mendelian randomization; OR, odds ratio; pQTL, protein quantitative trait loci; VD, visual disturbances.

A total of 1378 plasma proteins with *cis‐*pQTLs at a genome‐wide association level of *p* < 5 × 10^−8^ were included in the analyses. The discovery MR analyses identified 22 proteins (30 associations) that were associated with at least one outcome at a FDR‐corrected *p*‐value of <0.05. Among these 22/30 proteins/associations, 18/22 of them were shown to be significant in the validation MR analyses (Figure 2, Table 1 and Tables S5, S6). The MR Steiger test of directionality reinforced the validity of the assumption that exposures cause outcomes, as evidenced by significant *p*‐values for all associations observed in the MR analyses (Table S5).

**TABLE 1:** Associations significant in MR analysis.

Exposure	Outcome	Chr	No. of associations^a^	Tier	OR (95% CI)^b^	*p* Value	p_het^c^	p_ple^c^	p_HEIDI^d^	PP.H4.abf^e^	UK‐PPP/CSF^f^	Safety^g^
PNKP	Any migraine	19	1	2	1.312 (1.169, 1.472)	4.05 × 10^−6^	<0.01	0.03	0.09	0.99	NA/NA	No
CALCB	Any migraine	11	1	2	0.907 (0.869, 0.947)	9.80 × 10^−6^	0.04	0.28	<0.01	0.98	No/No	Yes
FER^h^	Any migraine	5	2	3	1.138 (1.075, 1.205)	9.35 × 10^−6^	0.04	0.01	0.01	0.96	NA/NA	No
ITIH4	Any migraine	3	3	1	0.862 (0.802, 0.926)	4.69 × 10^−5^	0.39	0.51	0.06	0.92	Yes/Yes	No
LRP11	Any migraine	6	4	1	0.968 (0.955, 0.981)	1.27 × 10^−6^	0.62	0.24	0.10	0.89	Yes/Yes	Yes
SERPING1^h^	Any migraine	11	2	3	0.959 (0.942, 0.976)	3.49 × 10^−6^	<0.01	0.18	<0.01	0.89	Yes/Yes	No
ITIH1	Any migraine	3	2	1	1.044 (1.024, 1.065)	1.08 × 10^−5^	0.78	0.85	0.65	0.87	Yes/NA	Yes
ADGRF5	Any migraine	6	2	1	0.964 (0.946, 0.982)	8.74 × 10^−5^	0.80	0.88	0.74	0.84	NA/Yes	No
PLG^h^	Any migraine	6	3	3	1.050 (1.024, 1.076)	1.59 × 10^−4^	0.47	0.50	0.30	0.30	Yes/No	No
INHBC	BRH	12	1	2	1.507 (1.282, 1.770)	6.08 × 10^−7^	0.23	0.95	0.01	0.98	No/Yes	No
PLCG1^h^	BRH	20	3	3	1.221 (1.105, 1.349)	9.30 × 10^−5^	0.00	NA	0.18	0.62	NA/NA	Yes
LRP11	BRH	6	4	2	0.969 (0.954, 0.984)	9.36 × 10^−5^	0.91	0.82	0.37	0.62	Yes/Yes	Yes
SERPING1^h^	BRH	11	2	3	0.952 (0.929, 0.975)	4.93 × 10^−5^	0.09	0.99	0.04	0.47	No/Yes	No
MRVI1	MO	11	3	2	0.800 (0.714, 0.896)	1.13 × 10^−4^	0.03	0.63	0.02	0.99	NA/NA	Yes
CHST4	MO	16	1	2	1.373 (1.190, 1.586)	1.52 × 10^−5^	0.01	NA	0.06	0.87	NA/NA	No
GP1BA	VD	17	1	2	0.811 (0.730, 0.900)	8.54 × 10^−5^	0.49	0.93	0.77	0.94	Yes/NA	No
ITIH1	VD	3	2	1	1.067 (1.031, 1.103)	1.61 × 10^−4^	0.23	0.18	0.73	0.89	Yes/NA	Yes
LRP11	VD	6	4	1	0.958 (0.936, 0.980)	2.14 × 10^−4^	0.41	0.62	0.11	0.83	Yes/Yes	Yes
SPINK7	VD	5	2	2	0.864 (0.800, 0.933)	1.89 × 10^−4^	<0.01	0.40	0.01	0.74	NA/NA	No
LGALS4	VD	19	3	2	0.859 (0.795, 0.929)	1.33 × 10^−4^	0.22	0.32	0.58	0.70	No/NA	No
ARHGAP25	VD	2	1	3	0.879 (0.822, 0.939)	1.42 × 10^−4^	0.79	0.50	0.11	0.34	Yes/No	Yes
ISOC1	VD	5	3	3	0.907 (0.862, 0.954)	1.57 × 10^−4^	0.67	0.59	0.77	0.06	NA/NA	No

Abbreviations: BRH, migraine with bad and recurrent headaches; CI, confidence interval; MA, migraine with aura; MO, migraine without aura; VD, migraine with visual disturbances; OR, odds ratio; HEIDI, heterogeneity in dependent instruments.

^a^ The number in each cell indicates the number of outcomes that are significantly or suggestively associated with the exposure in MR analysis. Definitions of significant and suggestive associations are provided in Figure 1.

^b^ The estimates were derived from the discovery MR analysis (*r* ^2^ = 0.001) using discovery protein samples. A *p*‐value of ≤2.14 × 10^−4^ corresponded to a false discovery rate *p*‐value of 0.05. The associations were further validated in the decode dataset, demonstrating a *p*‐value <0.05 and consistent direction of effect as observed in the discovery analysis. Additionally, except for the association between ARHGAP25 and VD, the results were confirmed using generalized summary‐data‐based MR or standard inverse variance‐weighted analysis using *r* ^2^ = 0.1 with linkage disequilibrium taken into account.

^c^ Heterogeneity and pleiotropy tests were conducted using the variants included in the generalized summary‐data‐based MR (or standard inverse variance‐weighted analysis) using the primary sample with a linkage disequilibrium *r* ^2^ = 0.1. If there are insufficient variants available, tests were performed by using the decode sample, if applicable. The Cochrane's *Q* test was used for heterogeneity testing, while the MR–Egger regression was employed for pleiotropy testing.

^d^ HEIDI test *p*‐values present here were results based on the discovery protein sample.

^e^ The maximum values of the posterior probability (PP.H4.abf) in co‐localization analyses were provided.

^f^ “Yes” indicates that the association was confirmed in the dataset with a *p*‐value <0.05 and with the same direction of effect as observed in the discovery analysis. For SERPING1 and LGALS4, although the corresponding associations were not confirmed under the *p*‐value threshold of 0.05 using the UK‐PPP data, the corresponding estimates were similar with the discovery estimates.

^g^ “Yes” indicates that there was no adverse consequences when modulating the proteins.

^h^ Except for these associations, all other associations has been confirmed by using variants (*r* ^2^ = 0.001) that were not associated with potential confounders identified by the PhenoScanner V2 database.

**FIGURE 3:**
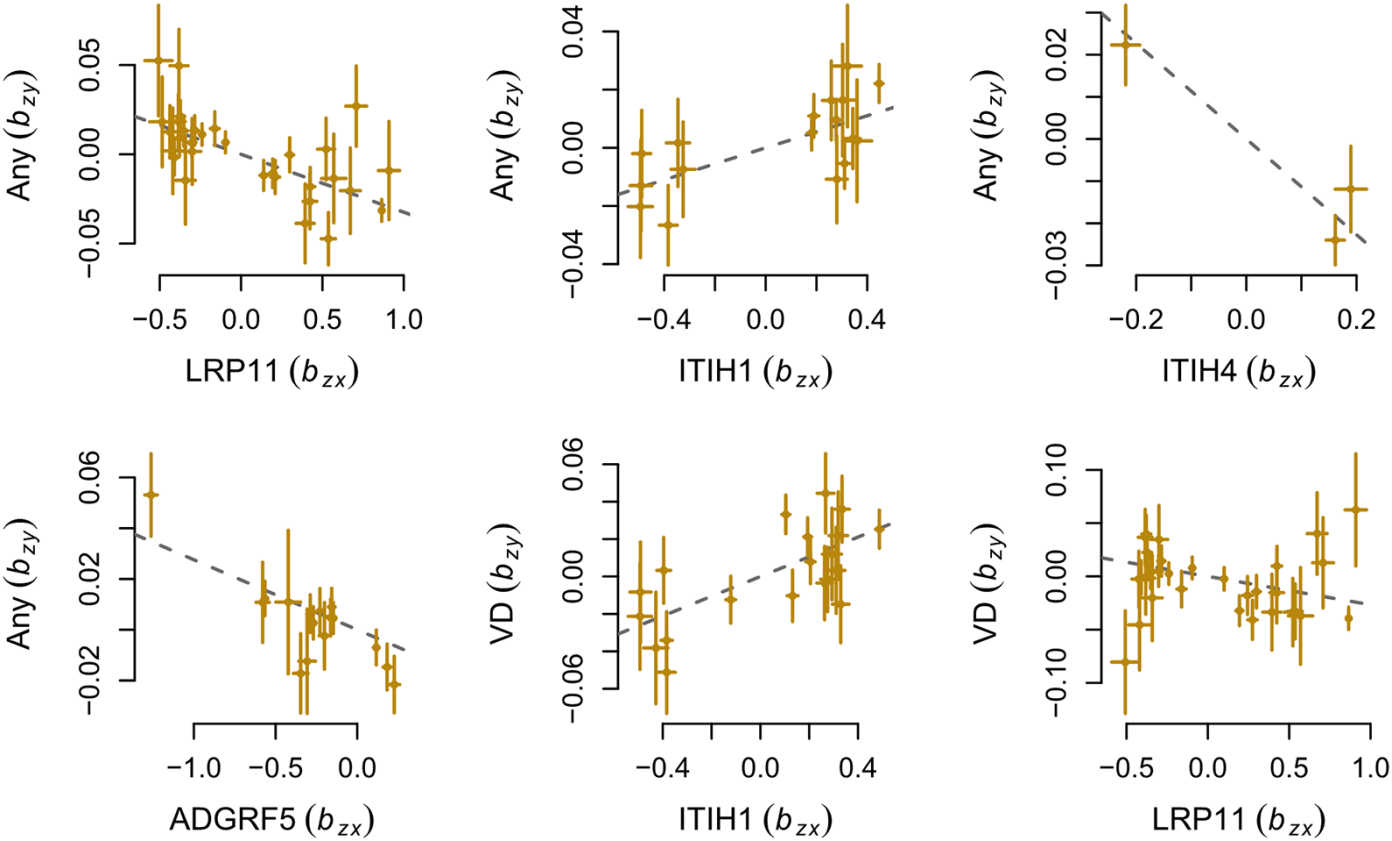
Scatter plots of tier 1 associations. Any, any migraine; VD, visual disturbances.

Using the GSMR/IVW method with a relaxed clumped *r* ^2^ threshold of 0.1 with linkage disequilibrium adjusted (Table 1, Tables S5–S8, Figure 3, Figures S1–S3), 18 out of the 22 associations significant in both the discovery and validation MR analyses were confirmed. One of the 22 associations failed to be confirmed, and 3 of the 22 associations did not have enough SNPs to be confirmed.

We next performed a sensitivity MR analysis using SNPs that were not associated with potential confounders identified by the PhenoScanner V2 database (Table 1 and Table S9). Seventeen out of the 22 associations were confirmed.

### 3.2 Bayesian co‐localization analysis

**FIGURE 4:**
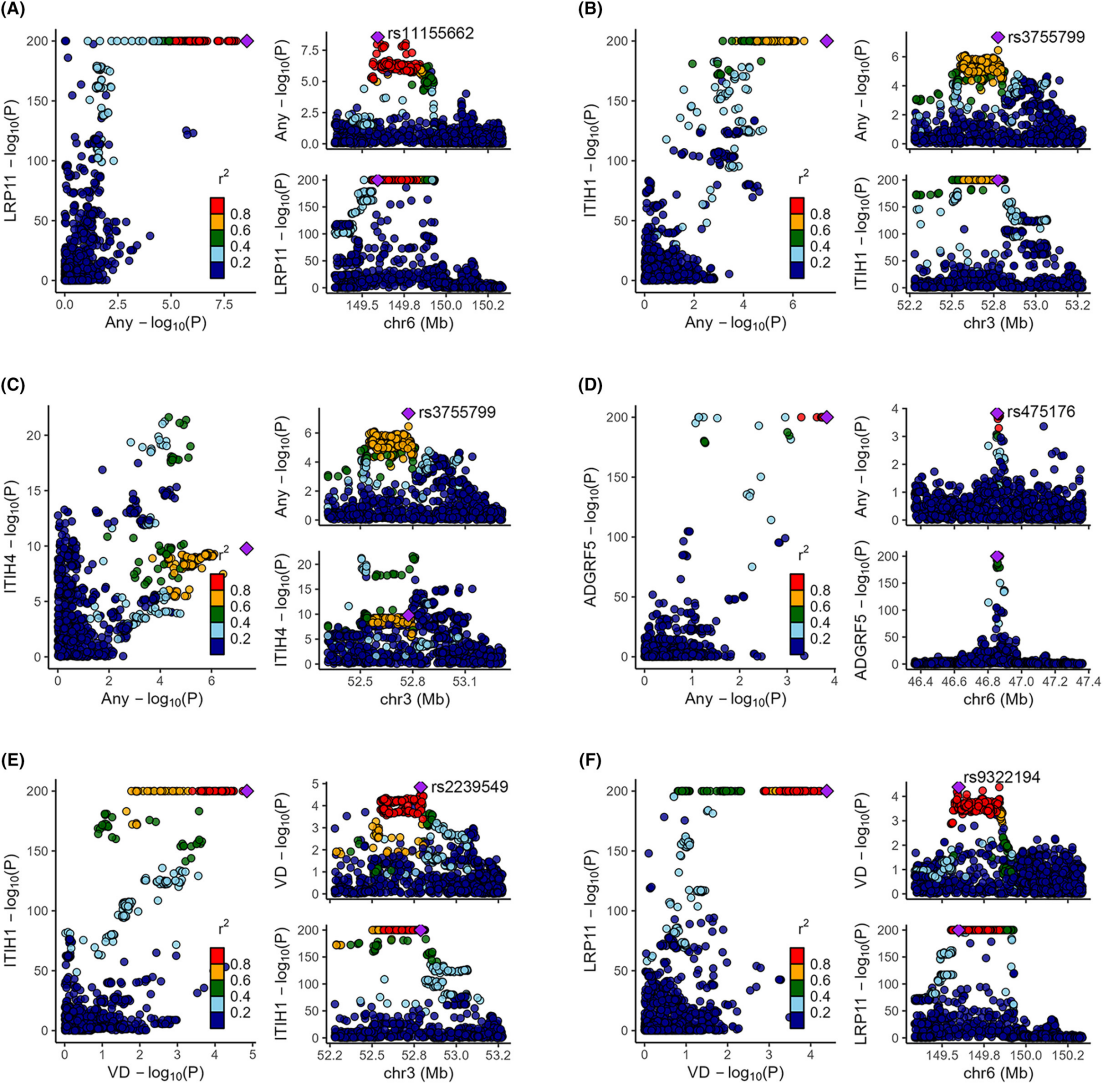
Co‐localization of plasma protein signals and migraine signals for tier 1 associations. (A) LRP11 and any migraine; (B) ITIH1 and any migraine; (C) ITIH4 and any migraine; (D) ADGRF5 and any migraine; (E) ITIH1 and migraine with visual disturbances; (F) LRP11 and migraine with visual disturbances.

Among the 22 significant associations determined by MR analysis, most of them were either strongly supported (PP.H4.abf > 0.8) or moderately supported (PP.H4.abf > 0.5) by co‐localization (Table 1 and Tables S5, S10, Figure 4, Figure S4). However, the associations between SERPING1 and BRH (PP.H4.abf = 0.47), ARHGAP25 and VD (PP.H4.abf = 0.34), PLG and any migraine (PP.H4.abf = 0.30), as well as ISOC1 and VD (PP.H4.abf = 0.06), did not receive sufficient support from co‐localization analysis, with a PP.H4.abf value below 0.5 (Table S5).

### 3.3 Associations tier classification

Based on our criteria (Figure 1), combined with the discovery and validation MR analyses, sensitivity analysis, heterogeneity tests, pleiotropy tests, the HEIDI test, and co‐localization analysis, six associations with four proteins that passed all of these analyses were assessed as tier 1 associations/proteins (Table 1 and Table S5). These associations demonstrated that LRP11 was significantly associated with a decreased risk of any migraine (OR = 0.968, *p* = 1.27 × 10^−6^) and VD (OR = 0.958, *p* = 2.14 × 10^−4^). ITIH1 was significantly associated with an increased risk of any migraine (OR = 1.044, *p* = 1.08 × 10^−5^) and VD (OR = 1.067, *p* = 1.61 × 10^−4^). Additionally, ITIH4 (OR = 0.862, *p* = 4.69 × 10^−5^) and ADGRF5 (OR = 0.964, *p* = 8.74 × 10^−5^) were significantly associated with a decreased risk of any migraine.

Another 9 significant associations were assessed as tier 2 associations. Furthermore, an additional 18 associations were assessed as tier 3 associations.

Potential reverse causal relationships were found for four tier 2 or tier 3 associations, but none were found for tier 1 associations (Table S11).

### 3.4 Compare with CSF pQTL and another plasma pQTL

**FIGURE 5:**
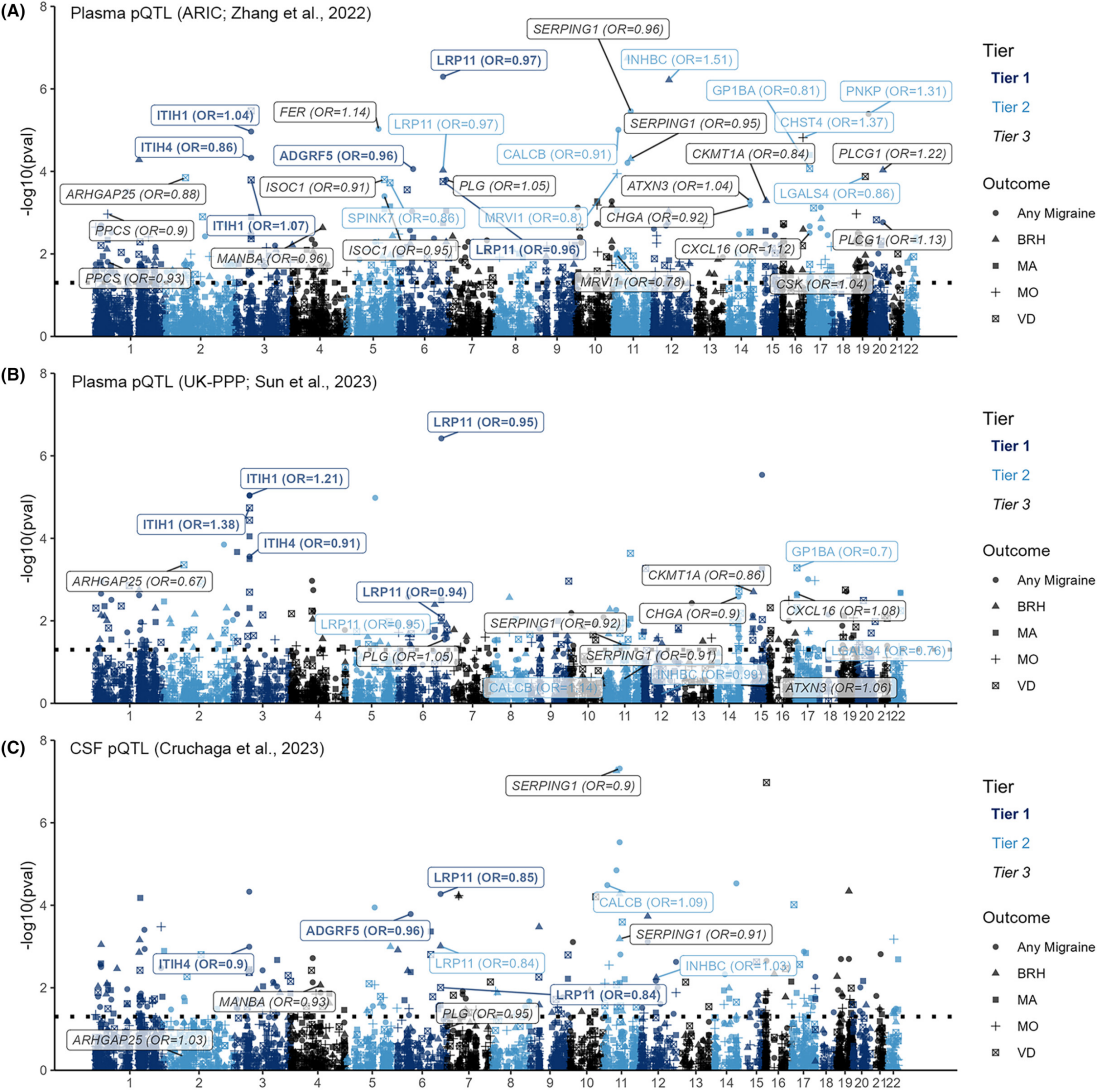
Comparison with MR analyses using pQTLs from the UK‐PPP with the Olink platform and using CSF pQTLs. (A) The MR results using discovery pQTL data (ARIC; Zhang et al., 2022) with the SomaScan platform. (B) The MR results using pQTLs from the UK‐PPP with the Olink platform. (C) The MR results using CSF pQTL data with the SomaScan platform. Tier 1 associations are labeled with bold and dark blue text. Tier 2 associations are labeled with plain and light blue text. Tier 3 associations are labeled with italicized and black text. BRH, bad and recurrent migraine headaches; CSF, cerebrospinal fluid; MA, migraine with aura; MO, migraine without aura; MR, Mendelian randomization; OR, odds ratio; pQTL, protein quantitative trait loci; VD, visual disturbances.

We were able to perform the MR analysis using plasma pQTLs from the UK Biobank Pharma Proteomics Project (Olink platform) for five tier 1 associations, all of which showed similar effects compared with the discovery analysis (Table S12 and Figure 5). Additionally, we conducted MR analysis using plasma pQTLs from the UK Biobank Pharma Proteomics Project for five tier 2 associations. However, only two of them exhibited similar effects compared to the discovery analysis with a *p*‐value <0.05. For the associations of LGALS4 and VD, similar estimate were found but with a *p*‐value >0.05. Another eight tier 3 associations all showed similar estimates compared to the discovery analysis, although two of them had a *p*‐value >0.05.

Furthermore, we performed MR analysis using CSF pQTLs for four tier 1 associations, and all of them showed similar effects as in the discovery analysis (Table S13 and Figure 5). Two tier 2 associations and two tier 3 associations were confirmed (*p* < 0.05) using the CSF data.

Although plasma CALCB levels were negatively associated with migraine risk in the discovery analysis, CALCB levels were positively associated with migraine risk using CSF data. In addition, although not statistically significant, the estimate using plasma pQTLs from the UK Biobank Pharma Proteomics Project was opposite to the estimate in the discovery analysis.

Figure S5 depicts the Pearson correlation coefficients of protein–migraine associations across various pQTL datasets. The consistency of protein‐migraine associations is notably higher among datasets derived from the same platform compared to those from different platforms. Moreover, restricting the analysis to proteins with suggestive associations, as listed in Table S5, led to improvements in all correlation coefficients.

### 3.5 Protein–protein interaction, druggability evaluation, target safety assessments

When consider all of the 25 tier 1, tier 2, and tier 3 proteins, PPI found seven edges, which has significantly more interactions than expected number of 2 (PPI enrichment *p*‐value: 0.011). The associations were found between ITIH4 and ITIH1, ITIH4 and SERPING1, ITIH4 and PLG, PLG and ITIH1, PLG and SERPING1, PLG and CHGA, and ATXN3 and PNKP (Table S14). Functional enrichment analysis showed that some proteins are annotated with several particular terms.

In druggability evaluation, multiple approved drugs that target CALCB (calcitonin gene‐related peptide 2) for the treatment of migraines were identified (Table S15). We further found drugs targeted for another six proteins (CSK, FER, GP1BA, PLCG1, PLG, and SERPING1), although currently there are no indications for migraine.

In target safety assessments, MR analysis suggested that modulating tier 1 proteins of LRP11 and ITHI1 was not associated with safety concerns (Figure S6 and Table S16). However, modulating tier 1 protein of ADGRF5 was suggestively associated with an increased risk of ovarian cancer (*p*‐value of inverse variance‐weighted method = 0.047) and breast cancer (*p*‐value of inverse variance‐weighted method = 0.042). Modulating tier 1 protein of ITIH4 was suggestively associated with an increased risk of coronary heart disease (*p*‐value of Wald ratio method = 0.039). In addition, modulation of tier 2 proteins CALCB and MRV1, as well as modulation of tier 3 proteins PLCG1, ARHGAP25, CHGA, and MANBA, was not associated with safety concerns.

## 4 DISCUSSION

In this proteome‐wide MR study accompanied by co‐localization analyses, we utilized pQTL data from multiple sources and measurement platforms to identify causal proteins for migraine and its subtypes. We identified several proteins (LRP11, ITIH1, and ADGRF5) that were significant in both the discovery and validation MR analyses; all of them survived sensitivity analysis, heterogeneity test, and pleiotropy test. Additionally, these proteins were strongly supported by co‐localization analyses and showed no evidence of reverse causality. The corresponding genes have not been previously identified as causal genes in genetic studies. No evidence of potential adverse consequences was found when modulating plasma levels of LRP11 and ITIH1, but not ADGRF5. We also identified another 21 proteins that were suggestively associated with migraine risk. There is no evidence of potential adverse consequences when modulating the plasma levels for six of them (CALCB, MRV1, PLCG1, ARHGAP25, CHGA, and MANBA), and four of them (PLCG1, ARHGAP25, CHGA, and MANBA) were not reported by previous genetic studies.

Specifically, we found compelling evidence that LRP11 was associated with a decreased risk of both overall migraine and migraine with visual disturbances. Additionally, LRP11 showed suggestive associations with a decreased risk of two other migraine subtypes: migraine with aura and migraine with bad and recurrent headaches. We also found compelling evidence that ITIH1 was associated with an increased risk of both overall migraine and migraine with visual disturbances. Furthermore, we observed compelling evidence that ADGRF5 was linked to a decreased risk of overall migraine and suggestive evidence that it was associated with a decreased risk of migraine with aura. The three corresponding genes have not been identified in previous migraine GWASs,^27^, ^40^, ^41^, ^42^ nor have they been identified in previous studies using other genetic approaches aimed at identifying migraine causal genes by integrating migraine GWAS data with eQTL/pQTL data.^43^, ^44^

While ITIH4 was also associated with migraine risk, co‐localization analysis revealed that the causal variant (rs3755799) associated with both ITIH4 and any migraine is the same as the one linked to ITIH1 and any migraine. This variant (position: 52775177) is located closer to ITIH1 (location: 52777599 to 52792068) than ITIH4 (location: 52812962 to 52830672) and is associated with ITIH1 with a smaller *p*‐value compared to ITIH4 (<1 × 10^−200^ vs. 1.70 × 10^−10^). Previous studies have also shown that the expression of ITIH4 and ITIH1 may be co‐regulated, with inverse relationships between ITIH4 and ITIH1.^45^ Therefore, it is possible that ITIH4 may not be the true causal protein.

LRP11, low‐density lipoprotein receptor‐related protein 11, is a member of the low‐density lipoprotein receptor‐related protein family. These transmembrane proteins play a crucial role in regulating cholesterol homeostasis through receptor‐mediated endocytosis of lipoprotein particles.^46^ Prior studies have indicated that LRP11 might be involved in cervical cancer and prostate cancer progression by increasing cell viability and accelerating the cell cycle.^47^, ^48^ This also might be partly attribute to the negative correlation between LRP11 expression and the infiltration levels of various immune cells, including CD8 T cells, NK cells, and gamma delta T cells.^49^ However, our MR analysis did not found LRP11 was positively associated with cervical cancer or prostate cancer (*p* values <0.05). On the contrary, we found suggestive evidence that LRP11 was negatively associated with lung cancer (OR = 0.970, *p* = 0.003). While evaluating the druggability potential of LRP11 using the Open Targets Platform (https://www.opentargets.org/), we observed a genetic correlation between LRP11 and circulating C‐reactive protein levels. Subsequent MR analysis confirmed a negative association between LRP11 and circulating C‐reactive protein levels (beta = −0.013, *p* = 7.41 × 10^−8^) (Table S17). Our analyses indicate that LRP11 could be a promising target without significant adverse consequences and may be linked to low‐grade inflammation.

ITIH1, inter‐alpha‐trypsin inhibitor heavy chain H1, is a member of the inter‐alpha‐trypsin inhibitor protein family. ITIH1 and ITIH2 can be covalently attached to bikunin (also known as ulinastatin), forming the inter‐alpha‐trypsin inhibitor complex.^50^ The roles of this complex include stabilizing and constructing extracellular matrix tissues, controlling neutrophil activation, acting as a plasmin inhibitor, inhibiting complement activation during systemic inflammation, and inhibiting hyaluronidase.^50^, ^51^ Additionally, studies have shown that inter‐alpha‐inhibitor proteins exert neuroprotective effects following exposure to hypoxic–ischemic conditions in neonatal rats.^51^ In contrast to the above evidence, our focus is on ITIH1 itself, and we found a positive association with migraine risk. Our findings are consistent with previous research indicating that ITIH1 polymorphisms are linked to an increased risk of schizophrenia and major depressive disorder,^52^ as well as cognitive dysfunction in psychotic disorders.^53^ We confirmed that alleles associated with an elevated risk of these conditions were related to decreased ITIH1 levels in the ARIC data. Furthermore, our MR analysis confirmed the causal effects of increased ITIH1 levels on schizophrenia (OR = 1.105, *p* = 1.24 × 10^−15^) and major depressive disorder risk (OR = 1.029, *p* = 9.26 × 10^−5^) (Table S17). These findings suggest that ITIH1 could be a promising target for multiple neuropsychiatric disorders.

ADGRF5, adhesion G protein‐coupled receptor F5, is a member of the adhesion G protein‐coupled receptor family and is highly expressed in the lung and kidney.^54^ It plays a critical role in lung surfactant homeostasis.^54^ ADGRF5 was also found to be expressed in adipose tissue and may play a beneficial role in glucose tolerance and insulin resistance.^54^ However, it has been suggested that ADGRF5 may be a potent regulator of cancer progression.^55^ In target safety assessments, MR analyses found that increased ADGRF5 levels were suggestively associated with an increased risk of ovarian cancer (*p*‐value of inverse variance‐weighted method = 0.047) and breast cancer (*p*‐value of inverse variance‐weighted method = 0.042). Therefore, the safety of targeting ADGRF5 for migraine should be further investigated.

This study has several strengths. First, we utilized plasma pQTL data from multiple sources and measurement platforms to identify the causal proteins for migraine and its subtypes. Second, we also conducted MR analyses using CSF pQTL data. Third, the largest publicly available migraine GWAS dataset was employed in this study. Additionally, we graded the proteins based on the number of outcomes identified for each protein. Fourth, multiple sensitivity analysis methods and co‐localization analyses were performed to enhance the primary findings. Firth, we also performed target safety assessments to assess the potential unintended adverse consequences when modulating the target.

This study also has several limitations. First, all the data we used were derived from individuals of European descent, which limits the generalizability of the findings to other populations. Second, the prevalence of migraine is significantly higher in females than in males. However, there was no data available for sex‐stratified analysis. Third, there was no brain pQTL data available at a certain significant threshold to validate the causal proteins identified in our study.

## 5 CONCLUSIONS

In this proteome‐wide MR study accompanied by co‐localization analyses, we utilized pQTL data from multiple sources and measurement platforms to identify the causal proteins for migraine and its subtypes. We identified several proteins (LRP11, ITIH1, and ADGRF5) that were significant in both the discovery and validation MR analyses; all of them survived sensitivity analysis, heterogeneity test, and pleiotropy test. Additionally, these proteins were strongly supported by co‐localization analyses and showed no evidence of reverse causality. The corresponding genes have not been previously identified as causal genes in genetic studies. No evidence of potential adverse consequences was found when modulating plasma levels of LRP11 and ITIH1, but not ADGRF5. We also identified another 21 proteins that were suggestively associated with migraine risk. There is no evidence of potential adverse consequences when modulating the plasma levels for six of them (CALCB, MRV1, PLCG1, ARHGAP25, CHGA, and MANBA), and four of them (PLCG1, ARHGAP25, CHGA, and MANBA) were not reported by previous genetic studies. Future studies are warranted to confirm the causal proteins and to investigate the underlying mechanisms.

## AUTHOR CONTRIBUTIONS

PPN analyzed and interpreted the data. RZ, CZ, and SL curated the data. PPN, RZ, CZ, and SL wrote the manuscript. YSL revised the manuscript. YSL supervised the study. All authors read and approved the final manuscript.

## FUNDING INFORMATION

This work was supported by the major project of medical science and technology in Henan Province under Grant [number SBGJ202101016].

## CONFLICT OF INTEREST STATEMENT

The authors declare that they have no competing interests.

## Supporting information


Figure S1.–S6.

Table S1.–S17.

## Data Availability

The supporting data for this study can be found in the supplementary files of the article as well as in the referenced studies within this article. The Plasma pQTL data from the Atherosclerosis Risk in Communities study were obtained from http://nilanjanchatterjeelab.org/pwas/. The Plasma pQTL data generated by Ferkingstad et al. were obtained from https://www.decode.com/summarydata/. The UK‐PPP plasma pQTL data were obtained from http://ukb‐ppp.gwas.eu. The CSF pQTL data were obtained from https://ontime.wustl.edu/. Summary data for migraine and its subtypes were obtained from https://www.decode.com/summarydata/.
