# Supplementary material for: Identifying novel proteins for migraine by integrating proteomes from blood and CSF with genome‐wide association data
Source: CNS Neurosci Ther. 2024 Jun 19;30(6):e14817. doi: 10.1111/cns.14817 (PMC11186850; doi:10.1111/cns.14817)
Supplement: Supplementary file 1 — Figure S1.–S6. [file CNS-30-e14817-s002.docx]

**Supplementary Figures**

Supplementary Figure 1 Forest plots of tier 1 associations

Supplementary Figure 2 Scatter plots of tier 2 associations

Supplementary Figure 3 Scatter plots of tier 3 associations

Supplementary Figure 4 Colocalization of plasma protein signals and migraine signals

Supplementary Figure 5 Heat maps show the Pearson correlation coefficients of protein-migraine associations across various pQTL datasets

Supplementary Figure 6 Potential adverse consequences when modulating the tier 1 targets


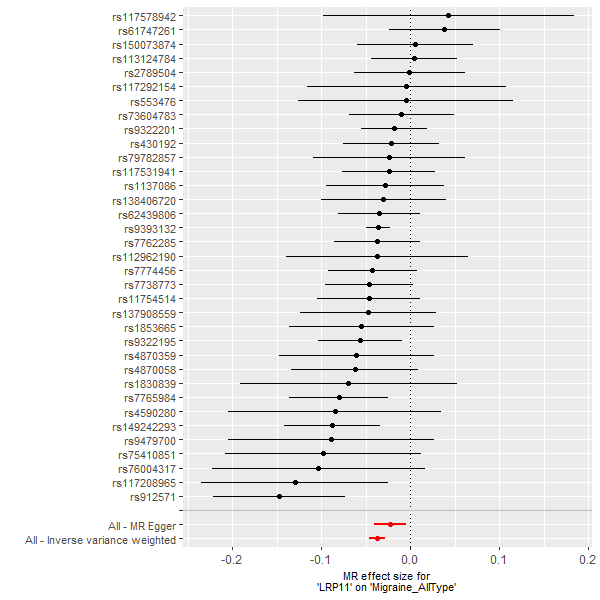


**Supplementary Figure 1a Forest plots of tier 1 associations**


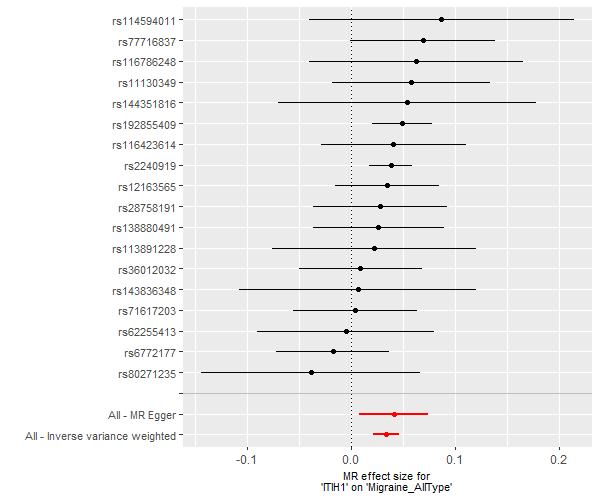


**Supplementary Figure 1b Forest plots of tier 1 associations**


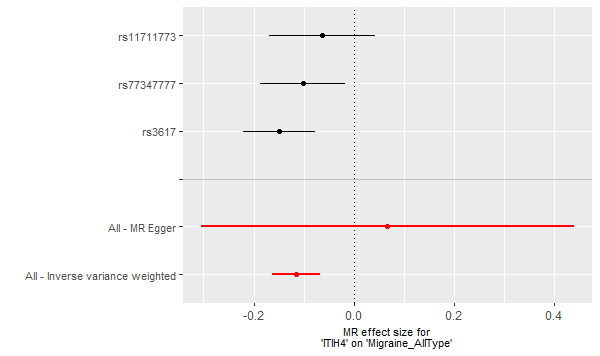


**Supplementary Figure 1c Forest plots of tier 1 associations**


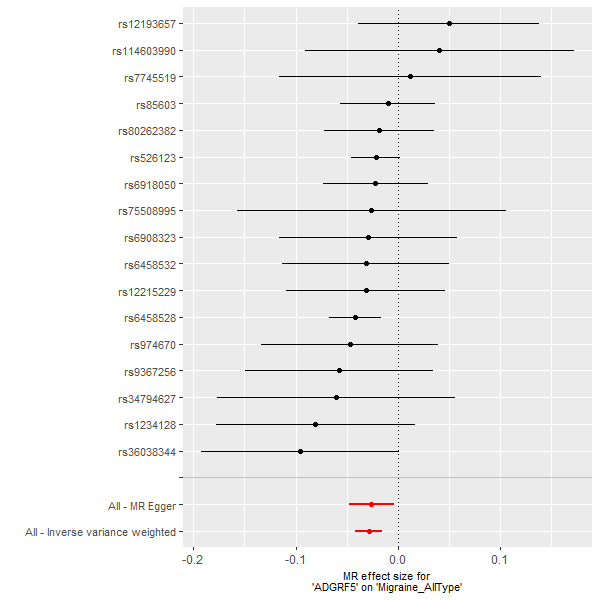


**Supplementary Figure 1d Forest plots of tier 1 associations**


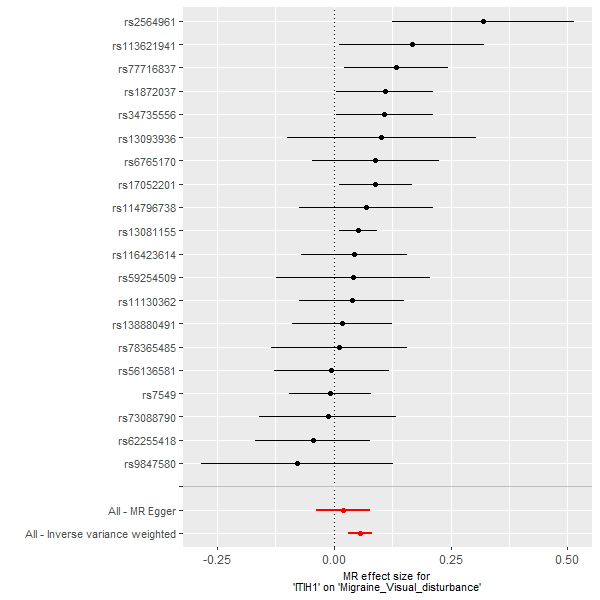


**Supplementary Figure 1e Forest plots of tier 1 associations**


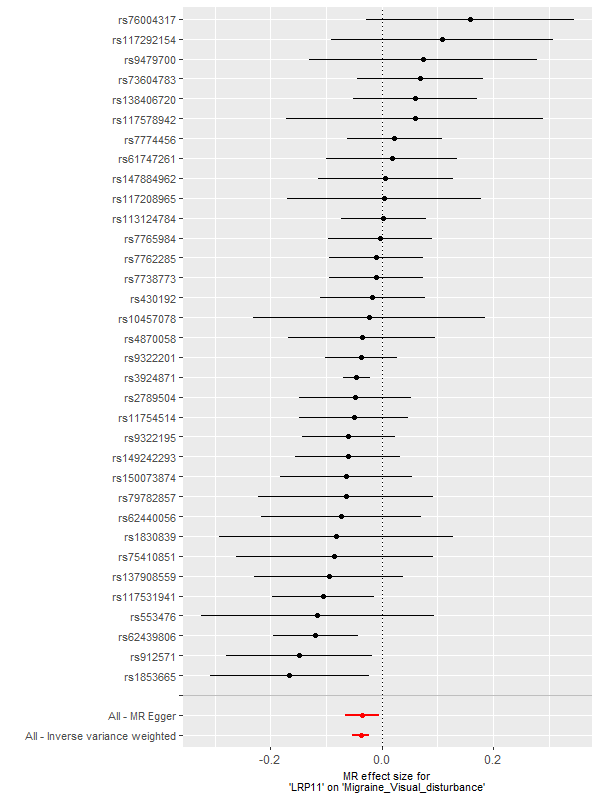


**Supplementary Figure 1f Forest plots of tier 1 associations**


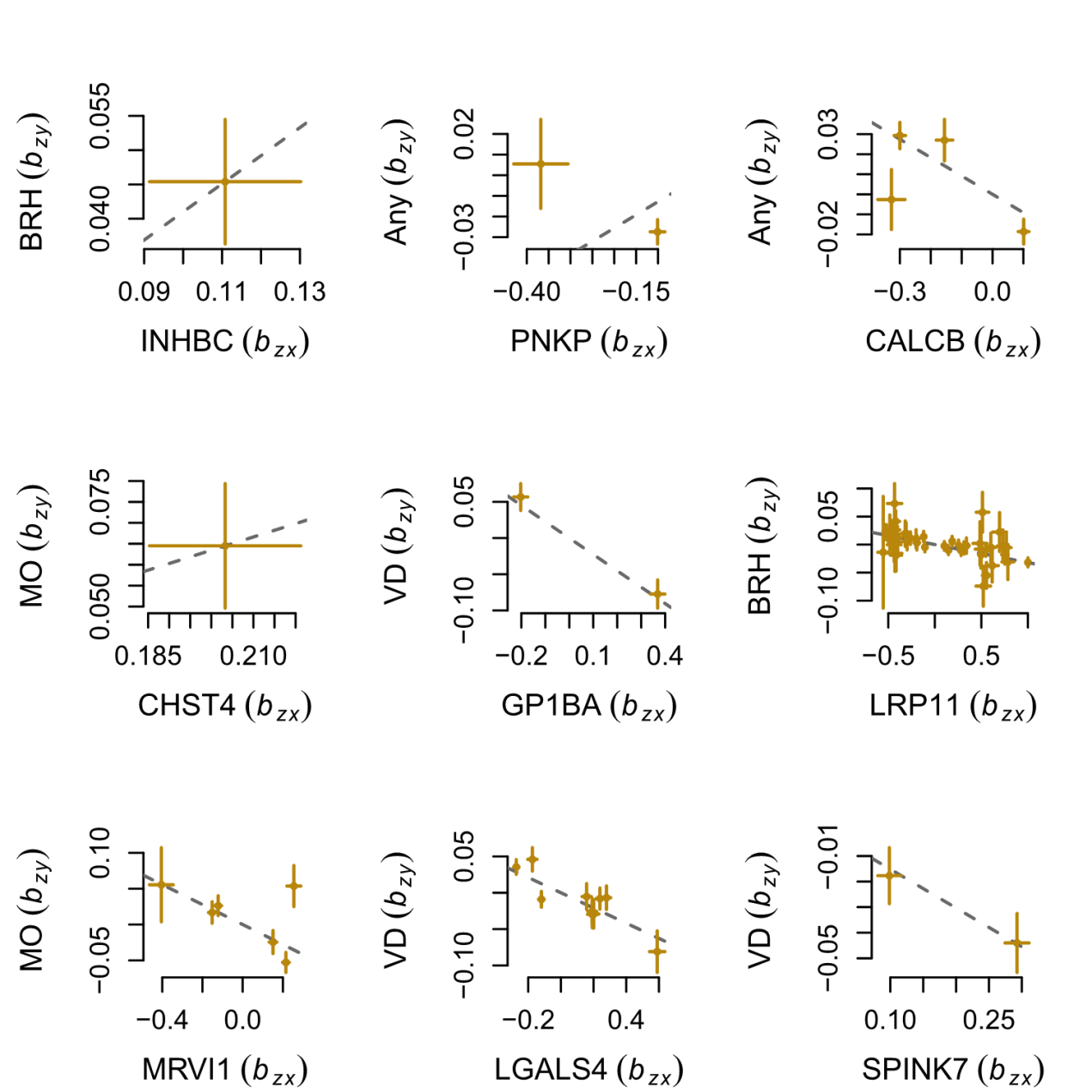


**Supplementary Figure 2 Scatter plots of tier 2 associations**


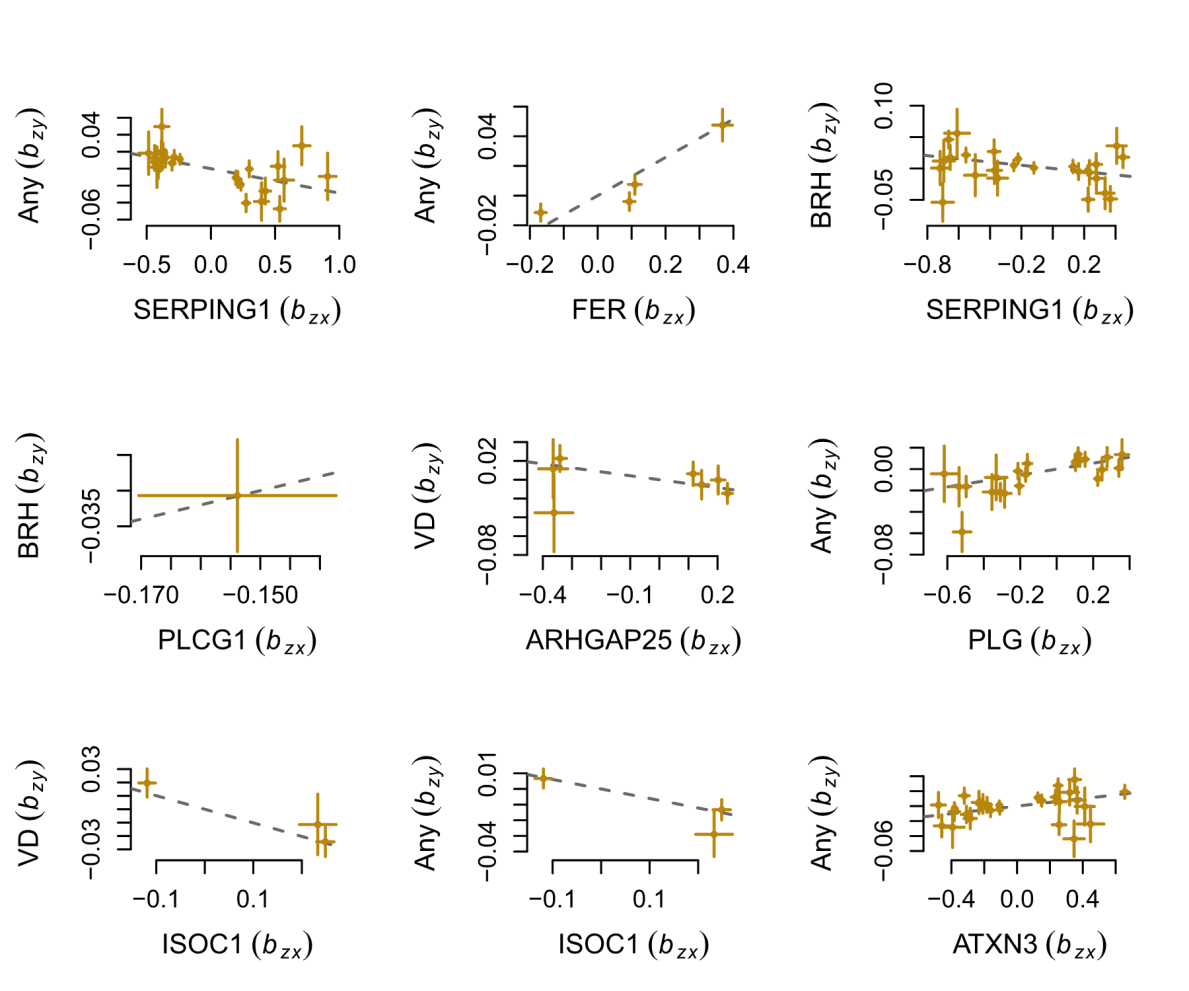


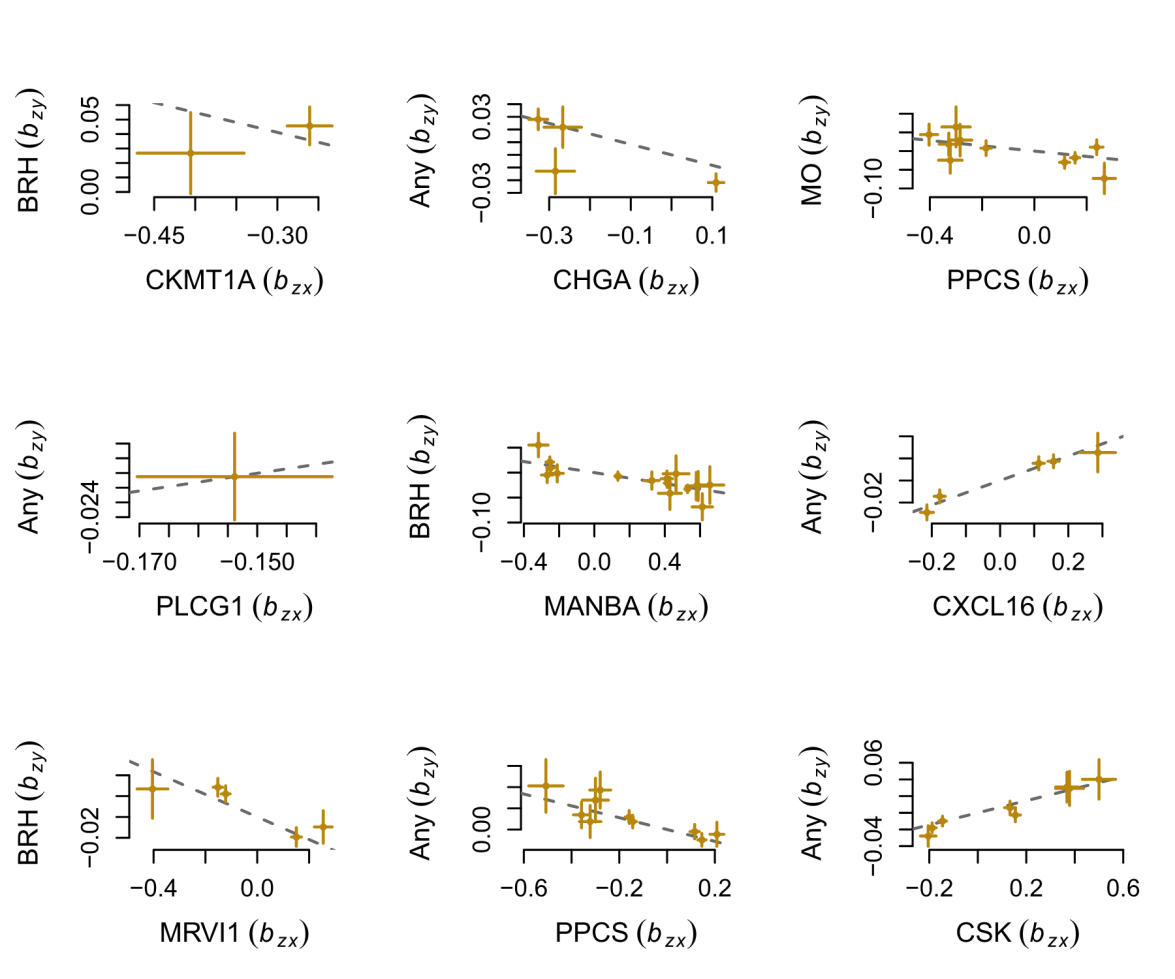


**Supplementary Figure 3 Scatter plots of tier 3 associations**


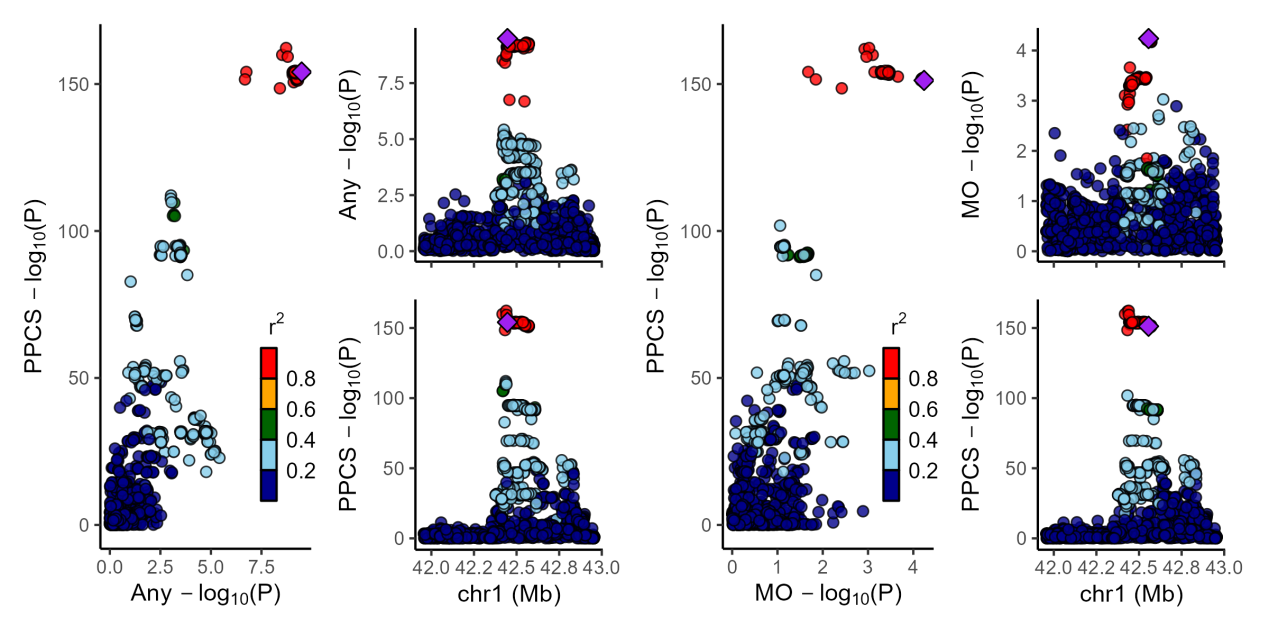


**Supplementary Figure 4 Colocalization of plasma protein signals and migraine signals (chr1)**


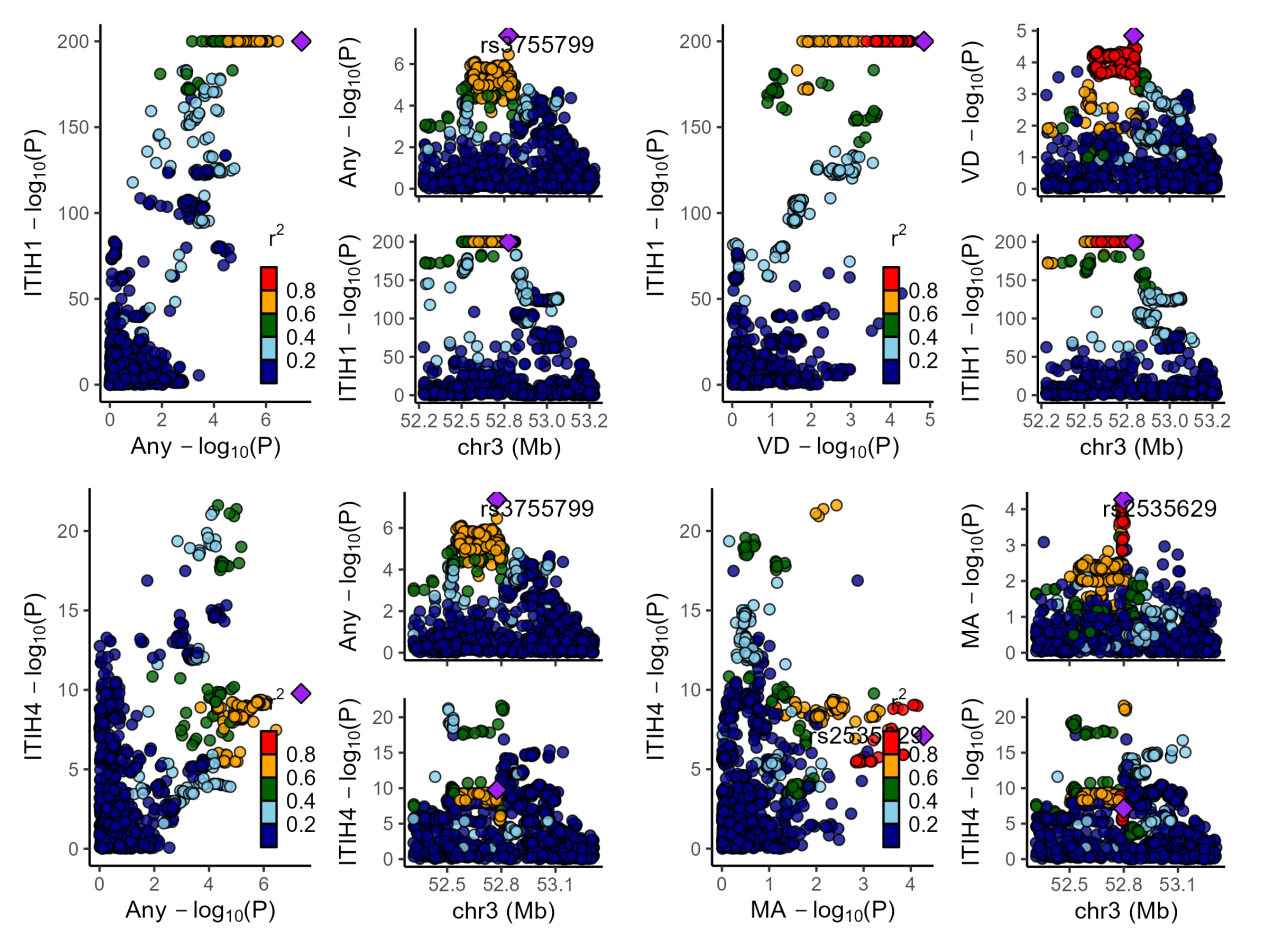


**Supplementary Figure 4 Colocalization of plasma protein signals and migraine signals (chr3)**


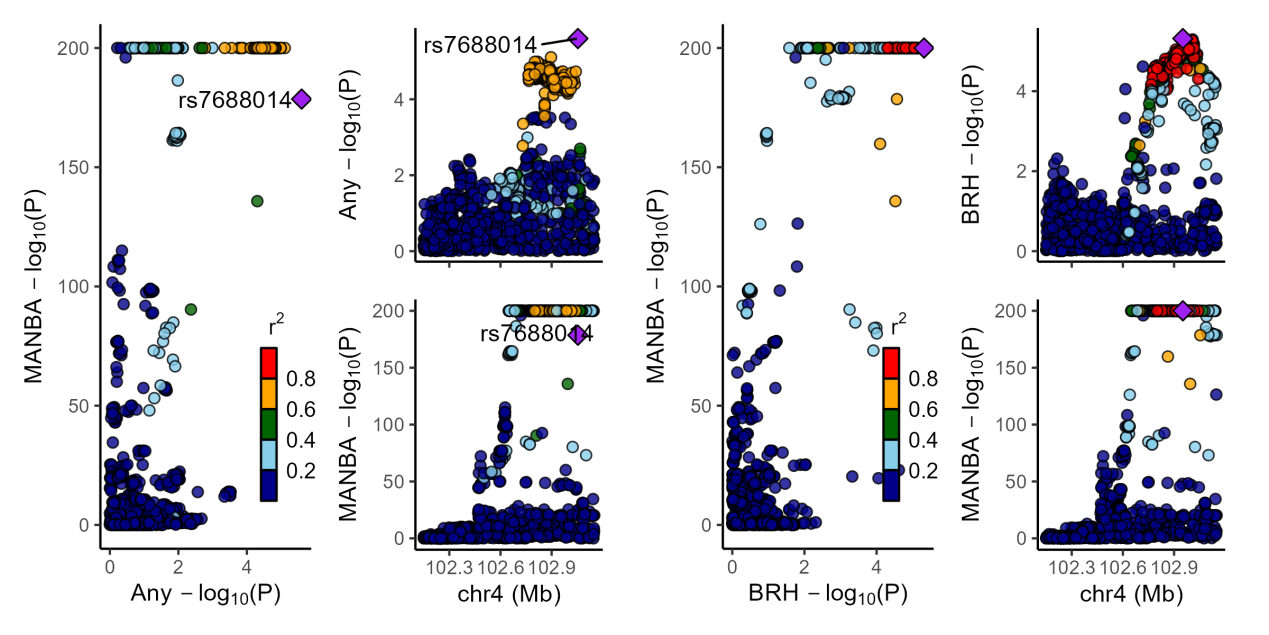


**Supplementary Figure 4 Colocalization of plasma protein signals and migraine signals (chr4)**


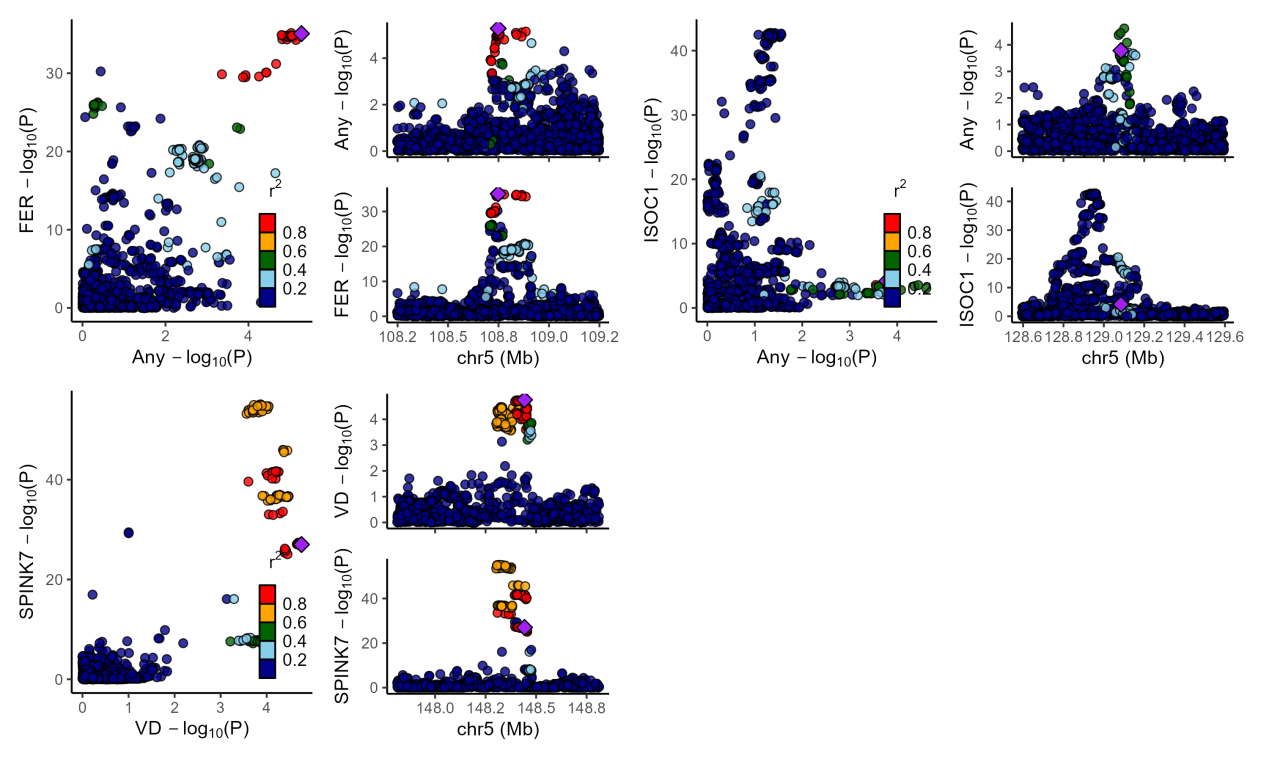


**Supplementary Figure 4 Colocalization of plasma protein signals and migraine signals (chr5)**


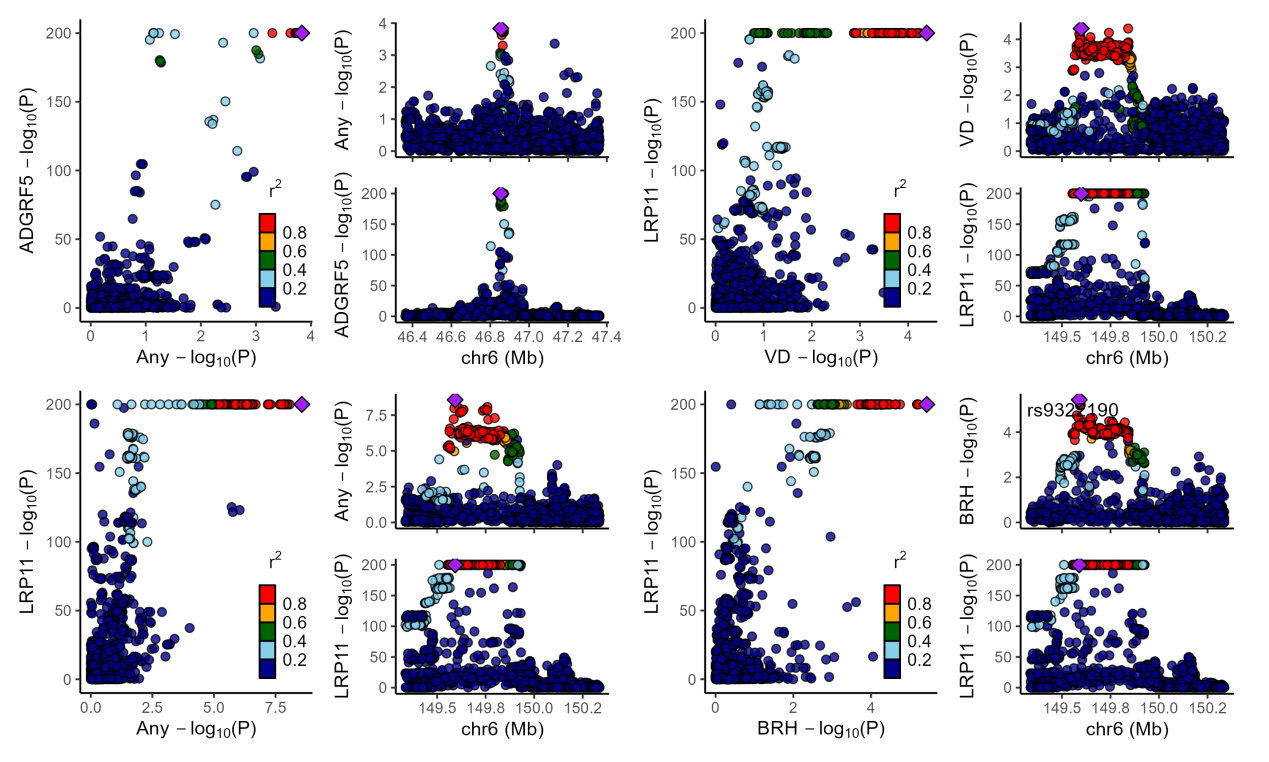


**Supplementary Figure 4 Colocalization of plasma protein signals and migraine signals (chr6)**


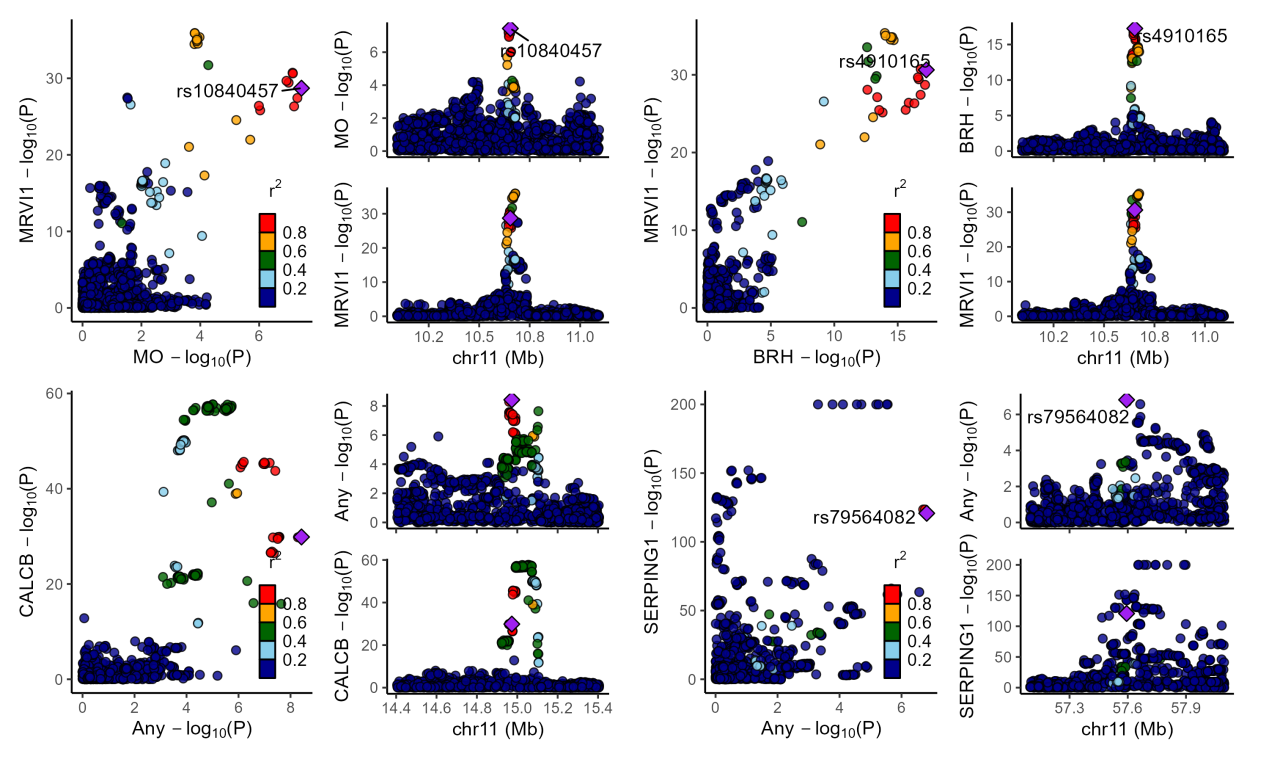


**Supplementary Figure 4 Colocalization of plasma protein signals and migraine signals (chr11)**


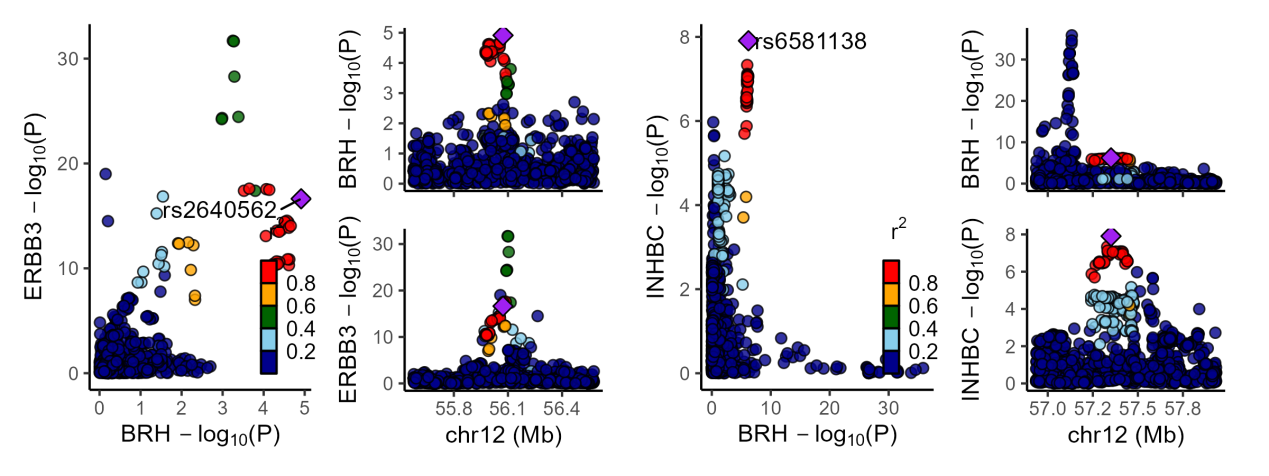


**Supplementary Figure 4 Colocalization of plasma protein signals and migraine signals (chr12)**


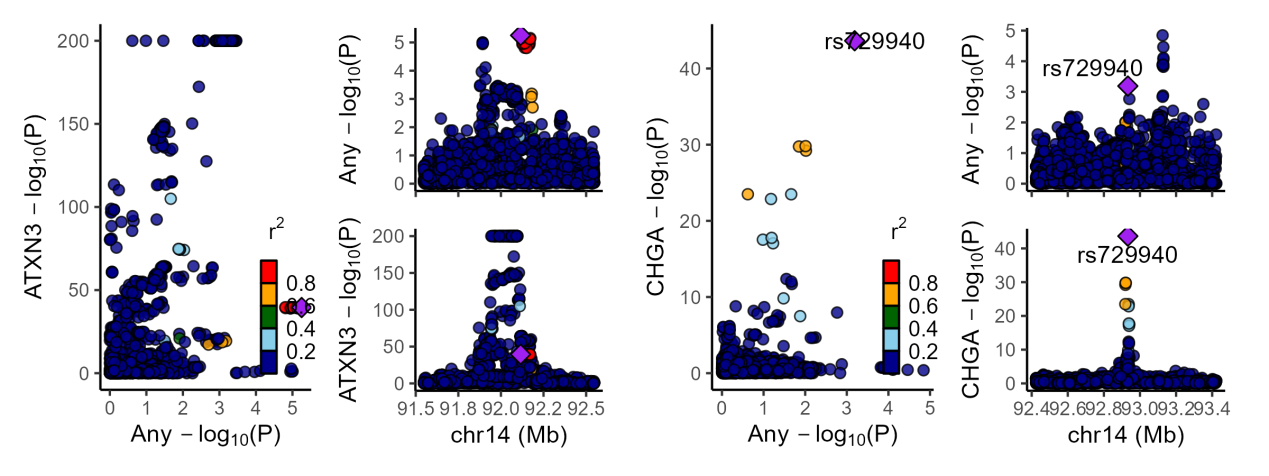


**Supplementary Figure 4 Colocalization of plasma protein signals and migraine signals (chr14)**


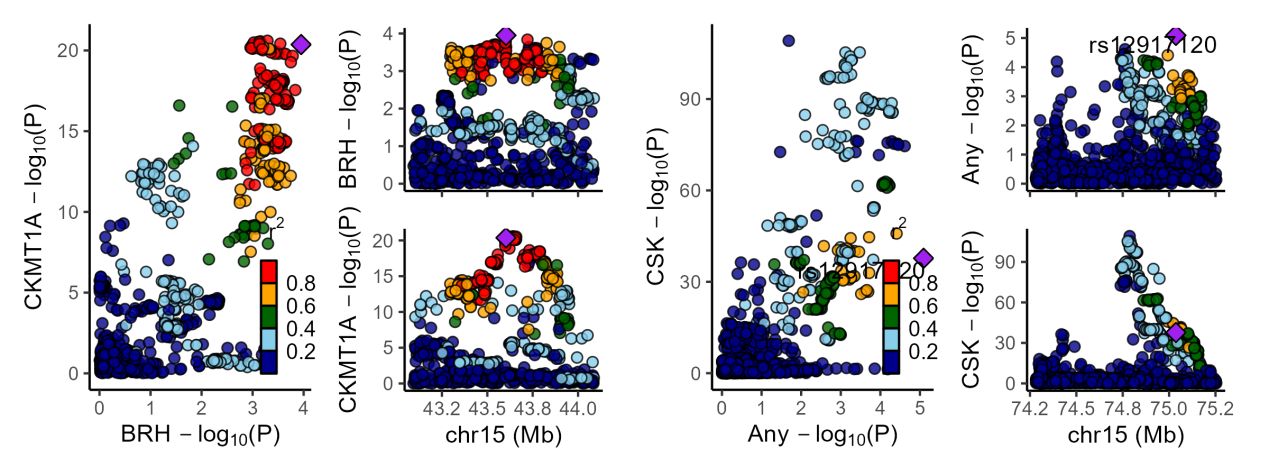


**Supplementary Figure 4 Colocalization of plasma protein signals and migraine signals (chr15)**


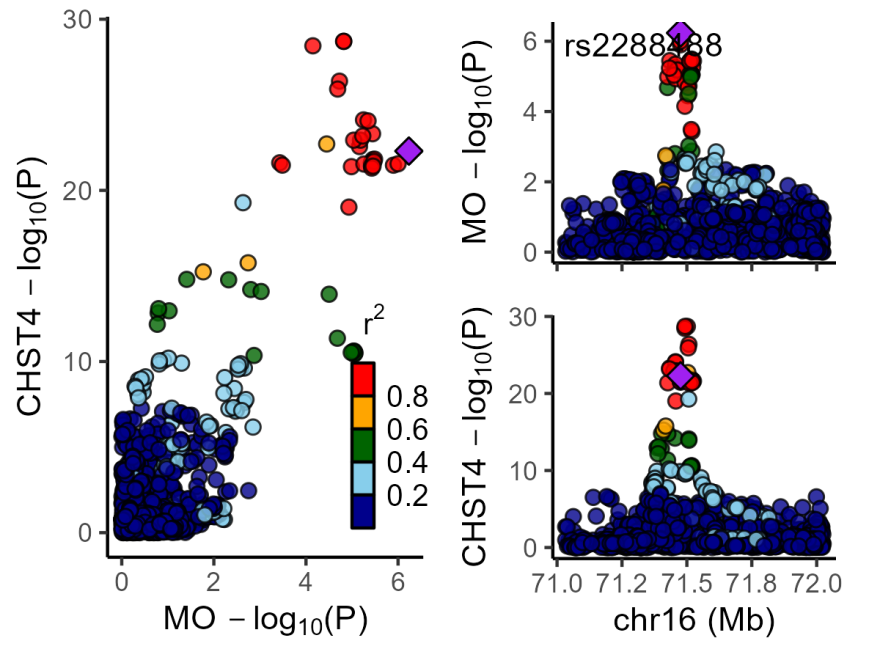


**Supplementary Figure 4 Colocalization of plasma protein signals and migraine signals (chr16)**


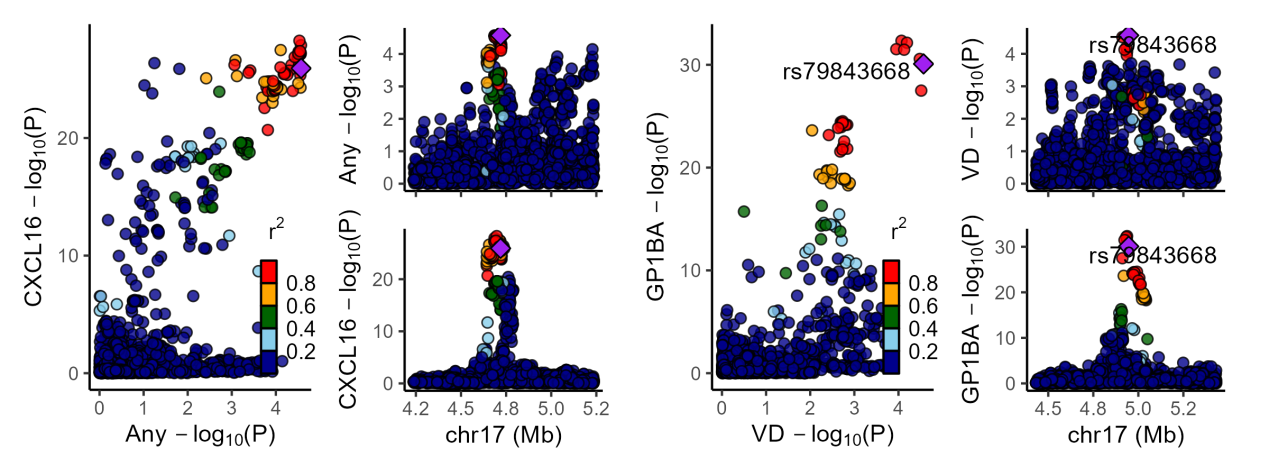


**Supplementary Figure 4 Colocalization of plasma protein signals and migraine signals (chr17)**


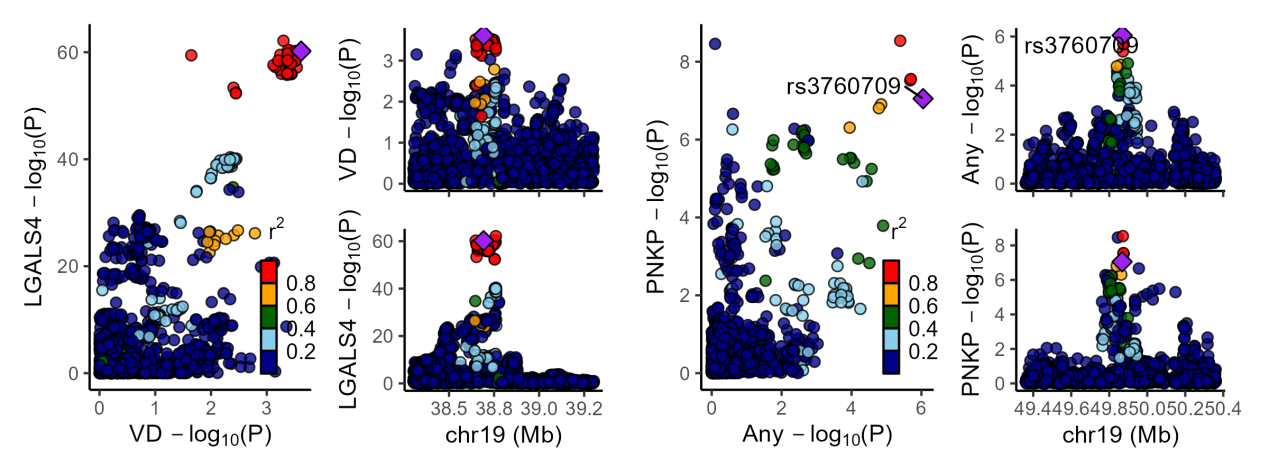


**Supplementary Figure 4 Colocalization of plasma protein signals and migraine signals (chr19)**


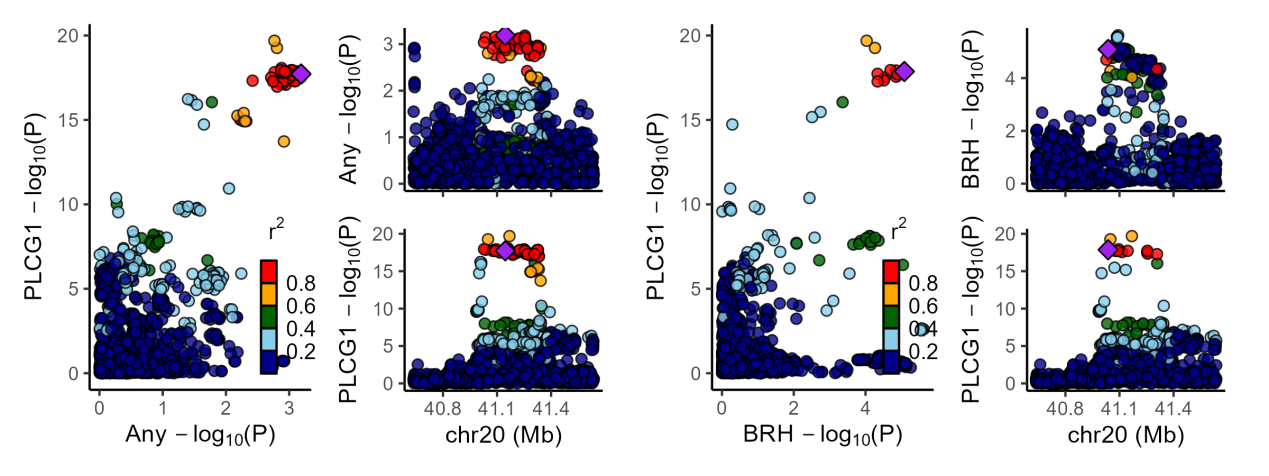


**Supplementary Figure 4 Colocalization of plasma protein signals and migraine signals (chr20)**


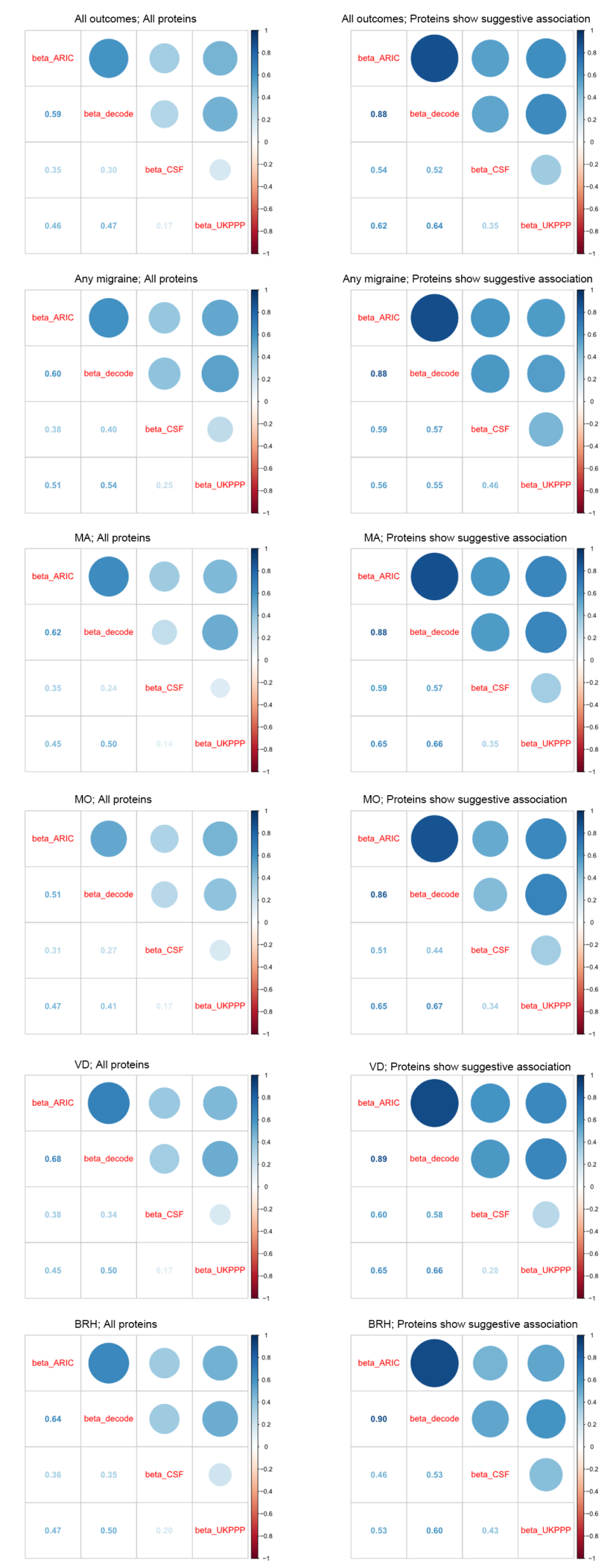


**Supplementary Figure 5 Heat maps show the Pearson correlation coefficients of protein-migraine associations across various pQTL datasets**

**
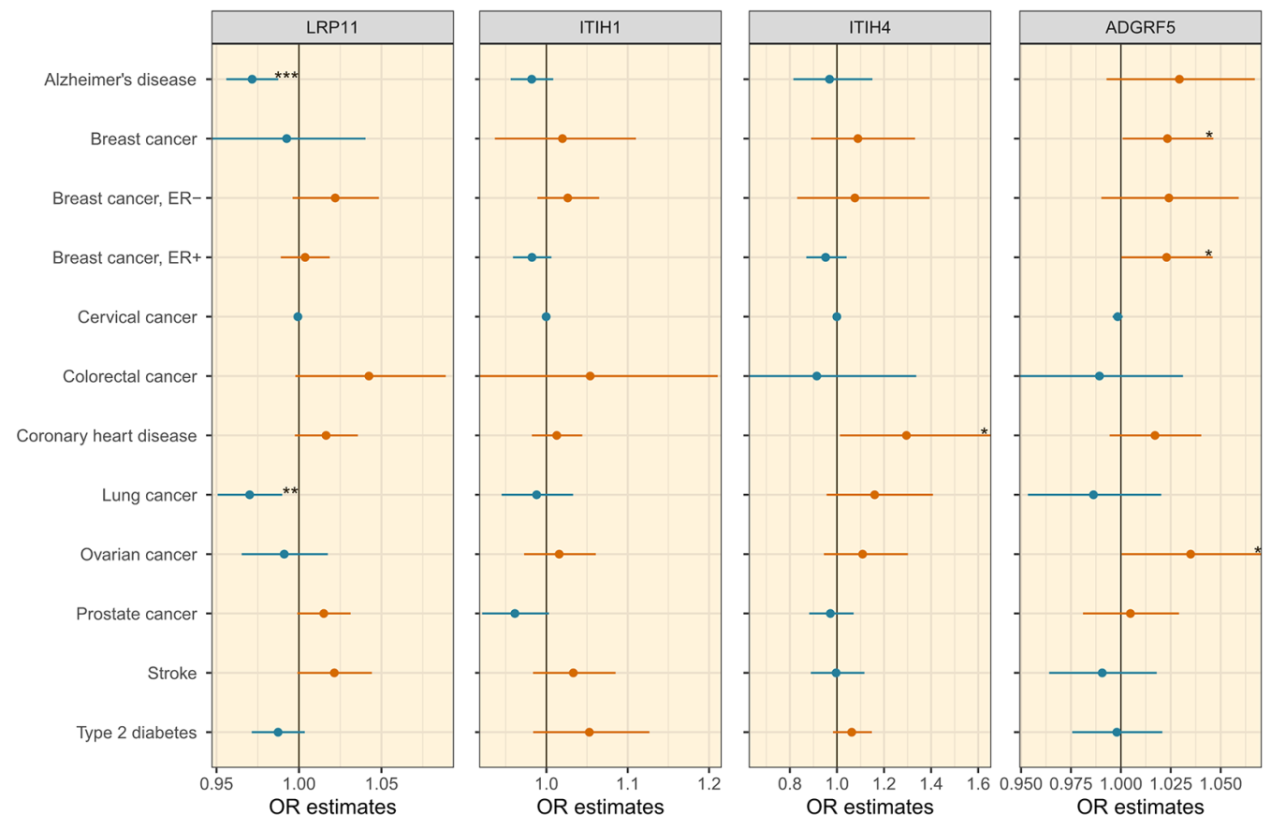
**

**Supplementary Figure 6 Potential adverse consequences when modulating the tier 1 targets**

For ITIH1, the odds ratio (OR) indicates the effect of a per unit decrease in ITIH1 plasma levels on the outcome; for other proteins, the OR indicates the effect of a per unit increase in plasma levels on the outcome. OR estimate > 1 indicates that modulating the target is associated with an increased risk of the corresponding outcome. Horizontal lines represent the 95% confidence intervals for odds ratios.

*indicates p-value < 0.05;

**indicates p-value < 0.01;

***indicates p-value < 0.001.
